# On Maximizing Energy and Spectral Efficiencies Using Small Cells in 5G and Beyond Networks †

**DOI:** 10.3390/s20061676

**Published:** 2020-03-17

**Authors:** Rony Kumer Saha

**Affiliations:** Radio and Spectrum Laboratory, KDDI Research Inc., 2-1-15 Ohara, Fujimino-shi, Saitama 356-8502, Japan; ro-saha@kddi-research.jp

**Keywords:** 5G and beyond, energy efficiency, framework, in-building, maximization, mobile communication, multiband, small cell, spectral efficiency

## Abstract

Addressing high capacity at low power as a key design goal envisages achieving high spectral efficiency (SE) and energy efficiency (EE) for the next-generation mobile networks. Because most data are generated in indoor environments, an ultra-dense deployment of small cells (SCs), particularly within multistory buildings in urban areas, is revealed as an effective technique to improve SE and EE by numerous studies. In this paper, we present a framework exploiting the four most interconnected-domain, including, power, time, frequency, and space, in the perspectives of SE and EE. Unlike existing literature, the framework takes advantage of higher degrees of freedom to maximize SE and EE using in-building SCs for 5G and beyond mobile networks. We derive average capacity, SE, and EE metrics, along with defining the condition for optimality of SE and EE and developing an algorithm for the framework. An extensive system-level evaluation is performed to show the impact of each domain on SE and EE. It is shown that employing multiband enabled SC base stations (SBSs) to increase operating spectrum in frequency-domain, reusing spectrum to SBSs more than once per building in spatial-domain, switching on and off each in-building SBS based on traffic availability to reduce SBS power consumption in power-domain, and using eICIC to avoid co-channel interference due to sharing spectrum with SBSs in time-domain can achieve massive SE and EE. Finally, we show that the proposed framework can satisfy SE, EE, as well as user experience data rate requirements for 5G and beyond mobile networks.

## 1. Introduction

### 1.1. Background and Motivation 

According to [1], most data is generated in indoor environments, particularly within buildings in urban areas. Moreover, due to increasing the use of rich multimedia services on mobile phones over time, the high data rate demand of users also increases. However, serving this large amount of data at a high rate with an outdoor macrocell base station (MBS) is difficult due to the presence of high external wall penetration loss of a building [2], the scarcity of available system bandwidth below 3-GHz [3], and a limit to the maximum transmission power to avoid excessive interference. An effective way to address this problem is to deploy small cell base stations (BSs) within buildings due to their small coverage and low transmission power such that an indoor UE can be served at a short distance and hence at low power. Since the transmit power and hence the coverage of a small cell base station (SBS) is small, in addition to the traditional 2-dimensional (2D) space in horizontal level, small cells can be deployed in vertical level (i.e., both intra-and inter-floor levels) of a building [4], resulting in an ultra-dense deployment of small cells [5] over a certain area of physical space of a macrocell.

Moreover, due to the existence of a high external wall penetration loss of a building, the same outdoor spectrum can be reused to indoor SBSs within a building by enforcing a proper interference management technique to SBSs such as time-domain almost blank subframe based (ABS) based enhanced inter-cell interference coordination (eICIC) technique [6]. Apart from the same operator’s spectrum, spectra from other systems, both homogeneous, i.e., another mobile network operator (MNO), and heterogeneous, e.g. a space-satellite system, can be shared dynamically with in-building SBSs. Furthermore, since in ABS based eICIC technique, victim BSs (i.e., SBSs) can serve their UEs’ traffic only during non-ABSs per ABS pattern period (APP), the discontinuous transmission (DTX) power mode [7] of SBSs can be made exploited such that the transmission power of an SBS can be switched off in absence of its UEs’ traffic requests, resulting in reducing network power consumption. Hence, based on the above discussion, it can be concluded that the capacity, and hence the spectral efficiency (SE), as well as the energy efficiency (EE) of a cellular mobile system, can be maximized by exploiting four most interconnected-domain, namely power, time, frequency, and space.

### 1.2. Related Work and Problem Statement 

Achieving high network SE is considered as one of the major traditional approaches to designing mobile networks. Accordingly, extensive studies, e.g., [8,9,10,11,12], have already addressed the SE issue of mobile networks. However, recently, environmental protection and energy-saving have also become potential issues raised by global communities, resulting in asking researchers for shifting focus towards energy-efficient network design approaches [13]. Consequently, numerous research studies, e.g., [13,14,15,16,17,18,19,20], have already addressed the issue of EE in mobile networks. Hence, due to increasing the energy consumption from large-scale deployment of small cells [21], in addition to SE, EE has also been considered as one of the key performance metrics for the design of future fifth-generation (5G) and beyond mobile systems [22,23,24]. Accordingly, several research works have already addressed both SE and EE together to investigate numerous critical concerns, including co-design framework, tradeoff, and joint optimization or maximization, of SE and EE.

For example, in [25] the authors have presented a multi-layer cooperative green heterogeneous network framework to significantly improve SE and EE in 5G communication systems. In [26], a co-design framework for SE and EE in the 5G mobile system perspective has been presented. Likewise, in [27], the authors have developed a statistical framework to quantify area SE and EE performance of a user-centric cloud-based radio access network for 5G mobile systems. Moreover, the authors in [23] have proposed an optimization framework for EE and SE maximization in a network where radio resources are shared among multiple operators in 5G.

The tradeoff between SE and EE has been investigated as well by several authors under various scenarios of networks, including 5G cognitive relay networks [28], Device-to-Device (D2D)-enabled uplink heterogeneous networks [29], downlink orthogonal frequency division multiple access networks [30], a hybrid satellite and terrestrial network from 5G perspective [31], and a cognitive satellite-vehicular network towards beyond 5G [32]. Similarly, the joint optimization of area EE and area SE for heterogeneous networks has been studied in [33,34]. Moreover, a few research studies have also been carried out toward maximizing SE and EE for numerous systems, including a wireless powered low latency non-orthogonal multiple access (NOMA) system in [35], an unmanned aerial vehicle (UAV)-enabled mobile relaying system in [36], and a full-duplex multiuser multiple-input multiple-output system in [37].

Likewise, many research works have focused on maximizing SE and EE, particularly for small cell-based heterogeneous networks. For example, the authors in [38] have considered joint power and admission control to maximize SE and EE for orthogonal frequency division multiplexing access based heterogeneous networks. In [39], the authors have explored potential cooperation gains via a cooperative bargaining game to counter the challenges of mitigating interference and saving energy to improve SE and EE. The authors in [40] have studied SE and EE performances of ultra-dense networks under different deployment strategies. In [41], the authors have proposed a novel bargaining cooperative game framework for energy-efficient and interference-aware power coordination in a dense small cell network to jointly address both SE and EE issues. Likewise, in [42] the authors have exploited distributed optimal cooperation and presented a utility maximization framework to jointly consider SE and EE in hyper-dense small cell networks. However, these existing research studies as aforementioned have considered exploiting either one or a combination of two to three domains of the four most interconnected-domain to limit the scopes of investigation to either SE, or EE, or both, which in turn results in not taking advantage of exploiting all four major domains mentioned in the previous section to maximize SE and EE even further.

### 1.3. Contribution and Limitation 

Hence, different from these aforementioned research studies, in this paper, we present a framework exploiting the four most interconnected-domain, including power, time, frequency, and space, due to being complementary to one another in the perspectives of SE and EE performance metrics of mobile communication systems. In contrast to the frameworks proposed in the existing literature, the presented framework takes advantage of higher degrees of freedom to maximize SE and EE performances using ultra-dense in-building small cells such that the expected envisaged SE and EE requirements for 5G and beyond cellular mobile systems can be achieved. In accomplishing so, the impact of enabling techniques in each of the four most interconnected-domain is investigated. More specifically, in frequency-domain, the framework exploits dynamic spectrum sharing of microwave spectrum in both terrestrial and non-terrestrial networks, as well as millimeter-wave (mmWave) spectrum in terrestrial networks, with multiband enabled in-building small cells to increase the amount of available operating spectrum, and hence SE, in indoor environments. However, to manage the co-channel interference (CCI) due to sharing the spectrum of one network to another in frequency-domain, the framework exploits the ABS based eICIC in time-domain.

To address EE, the framework considers minimizing the power consumption of SBSs by exploiting the DTX mode in SBS power in power-domain. Finally, the framework exploits the ultra-densification of SBSs in both horizontal (i.e., intra-floor) and vertical (i.e., inter-floor) levels of each multistory building in physical space-domain to increase the spatial reuse of spectrum to in-building small cells to increase both SE and EE. Note that, an increase in both SE and EE in space-domain can be clarified by the fact that an increase in spatial reuse of the same spectrum results in a corresponding increase in capacity. The increased capacity, in turn, increases SE for the same amount of spectrum (i.e., without licensing an additional spectrum), whereas increases EE due to the reduction in energy required to transmit per bit because of the low transmission power of a small cell. In doing so, we contribute the following in this paper:

- We first describe comprehensively the proposed framework in each domain followed by its system architecture.
- We perform mathematical modeling and analysis for the DTX power of SBS, 3-dimensional (3D) clustering of in-building SBSs and in-building small cells as a secondary system for dynamic spectrum sharing.
- We then derive the expressions for system-level average capacity, SE, and EE both with applying as well as without applying the DTX power mechanism as a function the number of multistory buildings per macrocell.
- The conditions for optimality for both SE and EE are also derived as a function of the number of multistory buildings per macrocell.
- We present an algorithm for the proposed framework and discuss default simulation parameters and assumptions that are used for the performance evaluation.
- An extensive performance evaluation is then carried out. The performance result for each of the four most interconnected-domain is discussed, as well as the conditions for optimality of a cost-efficient small cell deployment is defined.
- Finally, we compare the performances of the proposed framework in terms of SE, EE, as well as average user experience data rate with the corresponding requirements for 5G and beyond mobile systems. It is shown that the proposed framework can easily satisfy SE, EE, as well as average user experience data rate requirements for 5G and beyond mobile systems.

Note that, we limit the scope of the paper to the following in terms of, for example, parameters, assumptions, and features, to evaluate the performances of the proposed framework:

- We consider similar signal propagation characteristics of all SBSs located within the same building or different buildings.
- Any small cell is considered as enabled with four transceivers, each operating at a different frequency.
- Two microwave bands, namely 2-GHz and 3.5-GHz, and two mmWave bands, namely 28-GHz and 60-GHz, are considered for each small cell.
- The line-of-sight (LOS) model is considered for both mmWave bands due to high frequency and hence less multi-path effect, whereas the non-LOS (NLOS) model is considered for both microwave bands.
- 3D clustering of small cells per building is carried out by adopting [43] and CCI due to sharing the same spectrum with small cells is managed by adopting the ABS based eICIC technique.
- In modeling the DTX power mechanism, no switching delay due to transitioning between on-state and off-state, as well as no processing delay due to processing user traffic requests are considered.
- We consider Proportional Fair frequency-domain schedulers for all in-building SBSs.
- The performance evaluation is carried out mainly for in-building SBSs, whereas the performance comparison in terms of SE and EE of the proposed framework is carried out with that required for 5G and beyond mobile systems.

### 1.4. Paper Organization

The paper is organized as follows: In Section 2, the proposed framework and its system architecture are detailed. In Section 3, mathematical modeling and analysis, including DTX power of SBS, 3D clustering of in-building SBSs, and in-building small cells as a secondary system are discussed. In Section 4, system-level performance metrics for average capacity, SE, and EE with applying, as well as without applying, the DTX power mechanism to SBSs, in addition to the conditions for optimality for both SE and EE, are derived. Moreover, an algorithm for the proposed framework is also developed in Section 4. In Section 5, default simulation parameters and assumptions are discussed along with estimating indoor path loss for 28-GHz, as well as 3D cluster size and spatial reuse of spectrum per building. An extensive performance evaluation is carried out in Section 6 where performance results for each of the domains of four most interconnected-domain are discussed and the condition for optimality of a cost-efficient small cell deployment is defined. Moreover, the performances of the proposed framework in terms of SE, EE, as well as average user experience data rate with the corresponding requirements for 5G and beyond mobile systems are compared. The paper is concluded in Section 7.

## 2. Proposed Framework and System Architecture 

### 2.1. Proposed Framework

**Figure 1:**
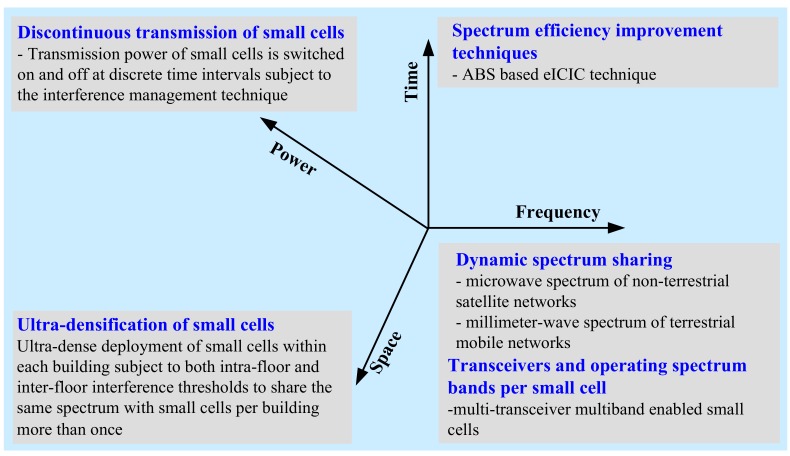
A proposed framework to maximize the capacity, EE, and SE of a mobile system using in-building small cells.

In Figure 1, we propose a framework to maximize the capacity, SE, and EE of a mobile system using in-building small cells. The framework exploits four domains, including power, time, frequency, and space, each of which we discuss in detail in what follows.

#### 2.1.1. Time-Domain Exploitation

Generally, when the same spectrum of system A is reused to BSs of another system B, CCI occurs, resulting in lowing the overall achievable capacity of both systems. An effective way to overcome CCI is not to serve user equipments (UEs) of both systems simultaneously such that there is a time orthogonality in serving UEs of both systems. This technique is referred to as time-domain ABS based eICIC developed primarily for the third generation partnership project (3GPP) Release 10 (i.e., fourth-generation (4G) mobile systems) [6] to avoid CCI and hence to improve overall system capacity and SE.

**Figure 2:**
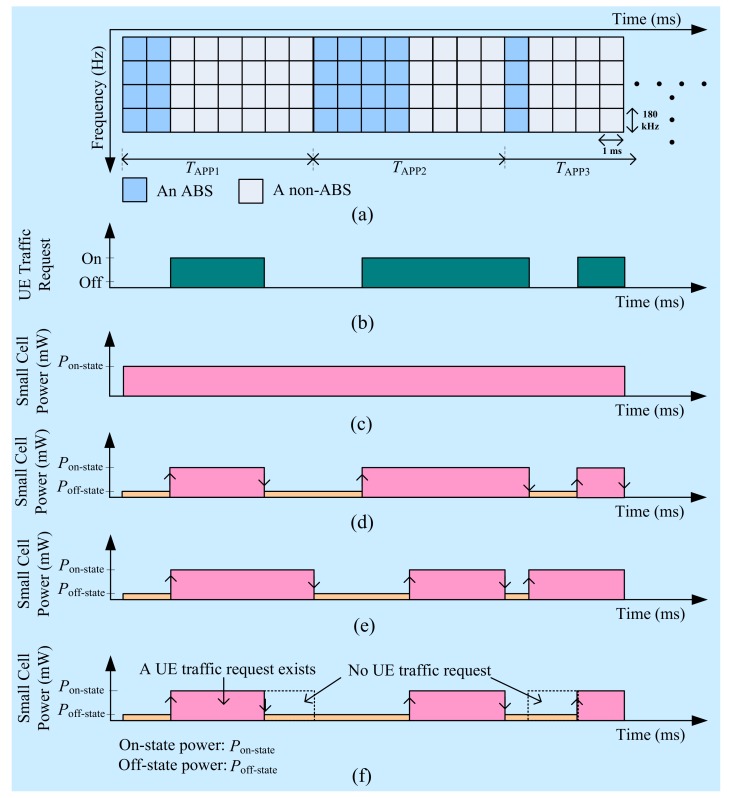
(**a**) an illustration for the time-domain ABS based eICIC technique for CCI management; (**b**) A UE traffic request over time; (**c**) without applying the DTX power mechanism to SBSs and the ABS based eICIC technique; (**d**) with applying the DTX power mechanism to SBSs and without applying the ABS based eICIC technique; (**e**) without applying the DTX power mechanism to SBSs and with applying the ABS based eICIC technique; (**f**) with applying the DTX power mechanism as well as the ABS based eICIC technique to SBSs.

In the ABS-based eICIC technique, an interfered BS is assigned to ABSs per APP so that other than the reference signals, no data and control signals can be transmitted at very low power to avoid CCI from interfering BSs. If in-building SBSs of a mobile system operate on the reused spectrum of another system, as BSs of a secondary system, they cause CCI with UEs of the primary system within their coverage in a building. In such a case, by applying the ABS-based eICIC technique with in-building SBSs, each SBS can serve its user data traffic only during non-ABSs so that UEs of the primary system can be protected by serving them only during ABSs as shown in Figure 2a.

#### 2.1.2. Power-Domain Exploitation 

Figure 2b shows an example traffic request of a UE to its serving SBS. If no power control mechanism is applied to the serving SBS, the transmission power of the SBS is always on as shown in Figure 2c, irrespective of whether or not the UE traffic request (Figure 2b) is available, resulting in unnecessary consumption of transmission power. However, if the DTX power mechanism is applied to the SBS, the transmission power of the SBS can be either reduced or switched to almost zero as shown in Figure 2d, resulting in avoiding the consumption of unnecessary transmission power of the SBS and hence improving EE of the system.

To enable the DTX power mechanism, an SBS can monitor to detect the status of its UEs’ traffic requests in every certain number of transmission time intervals (TTIs). Once an SBS senses that there is no more data traffic to serve, it can either reduce or switch to almost zero its transmission power and can remain so until there is a new traffic request from its UEs. To switch an SBS to the on-state from its off-state, a UE first sends a random access channel request to the SBS and continues communicating with the network through the SBS after exchanging several control-signaling messages with the SBS, e.g., radio resource control (RRC) connection initiation and complete message.

The same principle mentioned before can be explored for the ABS-based eICIC technique as well. For instance, Figure 2e shows an ideal condition for the ABS-based eICIC technique where it is assumed that an SBS always has a UE traffic request during non-ABSs such that the duration of the on-state power of the SBS varies with the duration of the non-ABSs (Figure 2a). However, in practice, an SBS may not always have a UE traffic request (Figure 2b) to serve. In such cases, by employing the DTX power mechanism to an SBS, the actual duration of the on-state power of the SBS can be reduced as shown in Figure 2f, resulting in improving EE of the system.

Note that, like the ABS-based eICIC technique, it is assumed that an SBS does not switch its transmission power completely to zero during an off-state due to exchanging some reference signals as shown in Figure 2. Moreover, for simplicity, we do not consider any switching delay to switch an SBS from the on-state to the off-state and vice versa. Hence, instead of keeping the transmission power of an in-building SBS always on, the application of the DTX power mechanism to each SBS can save unnecessary power consumption of SBSs leading to improving an overall system-wide EE.

#### 2.1.3. Frequency-Domain Exploitation

Dynamic spectrum sharing (DSS) is considered as one of the most effective techniques to address the spectrum scarcity issue of existing mobile networks. DSS takes advantage of the opportunistic use of the spectrum of a primary system to address the spectrum demand of a secondary system. More specifically, in time of no use of part of the licensed spectrum of one system, termed as a primary system, another system, termed as a secondary system, can use the corresponding part of the spectrum of the primary system subject to causing no interruption with ongoing services of the primary system. Since we focus on sharing spectrum with a terrestrial mobile system, the secondary system is by default a mobile system.

However, the primary system can be either the same type as the secondary system such as another terrestrial mobile system operating either at microwave frequency or at mmWave frequency or a different type of system from that of the secondary system such as a non-terrestrial satellite system. Moreover, the spectrum of the primary system can be either licensed (e.g., the dedicated spectra of a mobile system and a satellite system) or unlicensed (e.g., the spectrum of a WiFi system). Depending on the type of the primary system and its operating spectrum, numerous DSS techniques, including co-primary shared access (CoPSA) [44], co-channel shared access (CSA) [45], licensed shared access (LSA) [46], and licensed assisted access (LAA) [47], are proposed in the literature.

**Figure 3:**
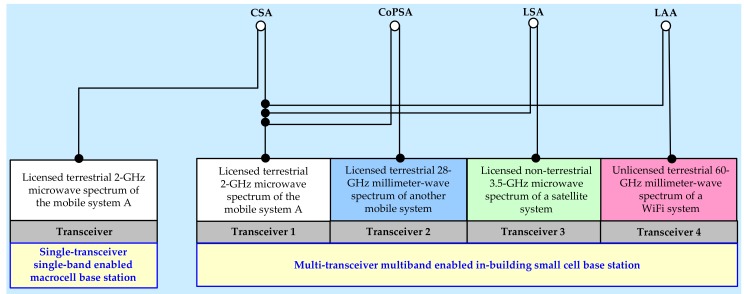
An illustration for a multi-transceiver multiband enabled SBS architecture of a licensed 2-GHz microwave terrestrial mobile system realizing DSS techniques, namely CSA, CoPSA, LSA, and LAA.

Furthermore, based on the number of transceivers and operating spectrum bands of an SBS [45] of a secondary mobile system, one or more of the above DSS techniques can be exploited simultaneously, resulting in increasing the amount of shared spectrum and hence the achievable capacity, SE, and EE of the secondary mobile system. Since the achievable capacity is directly proportional to the available spectrum, the amount of shared spectrum can be increased with an increase in the number of transceivers as well as their operating spectrum bands of an SBS of the secondary mobile system. In Figure 3, we present an illustrative realization of CoPSA by spectrum renting, CSA, LSA, and LAA where a primary system is respectively a terrestrial licensed 28-GHz mmWave mobile system, a terrestrial co-channel licensed 2-GHz microwave mobile system, a non-terrestrial licensed 3.5-GHz microwave satellite system, and a terrestrial unlicensed 60-GHz mmWave WiFi system (i.e., the WiFi standard IEEE 802.11ad) for a secondary terrestrial licensed 2-GHz microwave mobile system using its multi-transceiver multiband enabled in-building small cells.

#### 2.1.4. Space-Domain Exploitation

Network densification is considered as one of the major players to improve the capacity of next-generation mobile networks [5]. By reducing the coverage of cells, i.e., increasing the density of cells, the reuse of the same frequency can be increased over a given area of space. A small cell takes advantage of low transmission power and a short distance from its serving UE. In line with so, SBSs such as femtocell BSs (FBSs) are deployed as part of the macrocell in cellular mobile systems, particularly within multistory buildings in urban environments, such that the same outdoor macrocell spectrum can be reused to in-building small cells due to the presence of a high external wall penetration loss of a building. Since an increase in the number of times of reusing the same frequency over a given area of space increases correspondingly the overall network capacity and hence SE, it is recommended to reuse the same frequency to in-building small cells as many times as possible [2]. 

**Figure 4:**
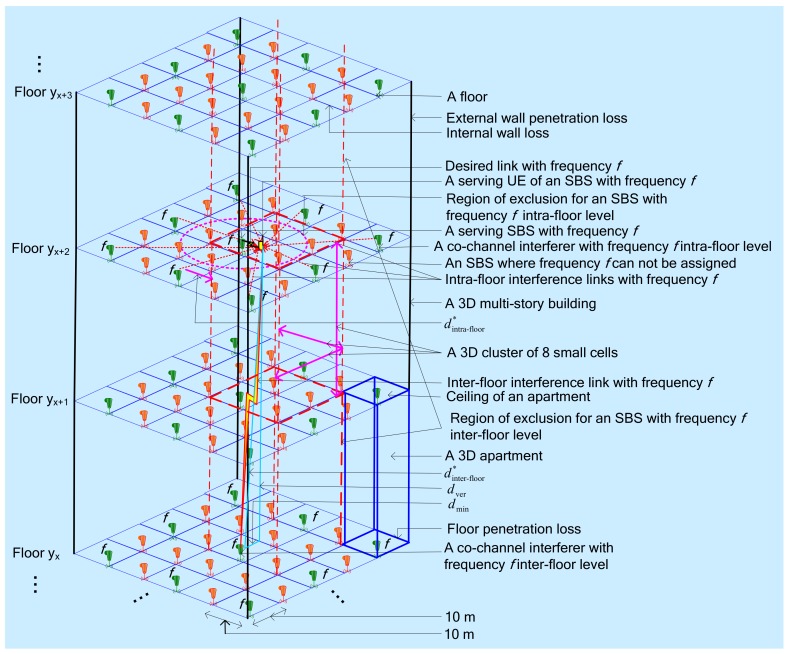
An illustration for an ultra-dense deployment of small cells in a 3D multistory building.

Note that, for a multistory building, small cells can be deployed on both intra-floor and inter-floor levels subject to satisfying a minimum distance between co-channel small cells, which are constrained by the allowable maximum level of CCI caused due to reusing the same spectrum in neighboring small cells [4]. Hence, in a 3D building, the density of small cells can be explored as well along with the vertical level, i.e., the inter-floor level of a multistory building, in addition to the traditional 2D horizontal space, i.e., the intra-floor level of a multistory building, resulting in an ultra-dense small cell deployment as shown in Figure 4. A set of small cells in both intra-and inter-floor levels constitutes a 3D cluster of small cell cube, and likewise, a group of 3D clusters can be formed per multistory building. The same frequency can then be reused to each 3D cluster of small cells. Hence, by increasing the number of 3D clusters per building, the number of times of reusing the same frequency, and hence the small cell network capacity and SE can be increased.

### 2.2. System Architecture 

**Figure 5:**
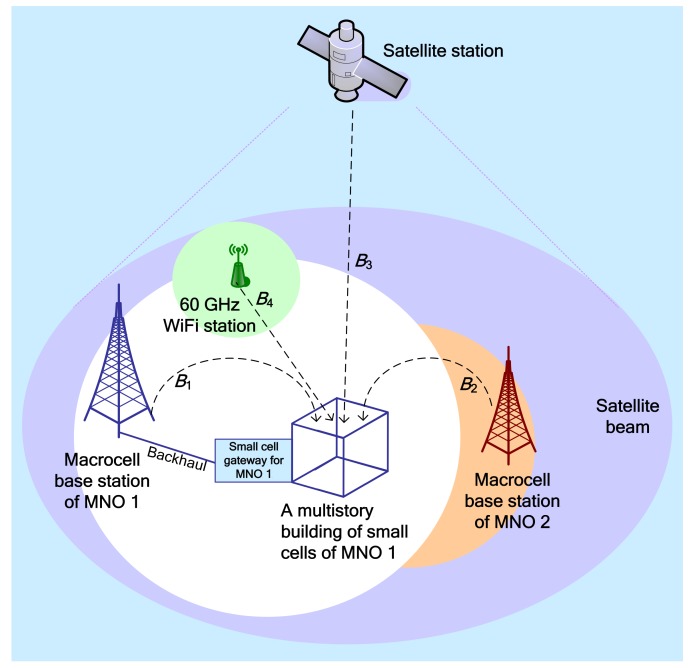
An illustration of the system architecture in the abstract level considered for the proposed framework.

The system architecture for realizing the proposed framework is shown in Figure 5. We assume that the capacity, SE, and EE of mobile network operator (MNO 1) need to be maximized such that being the secondary system, the enabling techniques of the proposed framework are applied to SBSs per 3D multistory building over the coverage of a macrocell of the secondary system, i.e., MNO 1. The deployment of SBSs within a building and the modeling of a multistory building follow Figure 4.

As discussed in Figure 3, CoPSA by spectrum renting, CSA, LSA, and LAA are realized with each SBS of MNO 1 per building by considering four primary systems, namely a terrestrial licensed 28-GHz mmWave mobile system for CoPSA by spectrum renting, a terrestrial co-channel licensed 2-GHz microwave mobile system for CSA, a non-terrestrial licensed 3.5-GHz satellite system for LSA, and a terrestrial unlicensed 60-GHz mmWave WiFi system, for the secondary terrestrial licensed 2-GHz microwave mobile system, i.e., MNO 1, using its multi-transceiver multiband enabled in-building small cells as shown in Figure 5. Note that, based on the discussion in the previous section, the proposed framework uses time- and power-domain to address mainly CCI and transmission power management (Figure 2), whereas space-and frequency-domain directly affect the achievable performance of the proposed framework. Since each of the primary systems contributes to a new DSS technique, the performance metric improves as the number of DSS techniques applied to SBSs per building increases.

For evaluating the secondary system, we assume that several outdoor macrocell UEs are offloaded to a set of picocells, and a certain percentage of macrocell UEs are considered within buildings deployed over the coverage of a macrocell of MNO 1. Further, we assume that whenever a macrocell UE of MNO 1 is within the coverage of an SBS in a building, it is offloaded to the corresponding SBS such that in place of the macrocell, the macrocell UE is then served by the in-building SBS of MNO 1 to avoid CCI with UEs of the SBS served by its transceiver 1. Moreover, we assume that a certain amount of spectrum of MNO 2 is rented exclusively by the MNO 1 such that no access to the corresponding spectrum of UEs of MNO 2 is made possible as long as the negotiation between MNO 1 and MNO 2 persists.

Finally, as shown in Figure 4, we assume that a multistory building consists of a set of regular square-grid apartments on each floor, and each building has multiple floors. We consider that an SBS is placed in the center of each apartment of a building and can serve at most one UE at any TTI. If each building has the same number of apartments each equipped with an SBS, the number of SBSs deployed per building is the same such that the number of 3D clusters of SBSs and hence the spectrum reuse gain from each building is also the same.

## 3. Mathematical Modeling and Analysis 

### 3.1. Modeling Discontinuous Transmission Power of Small Cell Base Stations 

**Figure 6:**
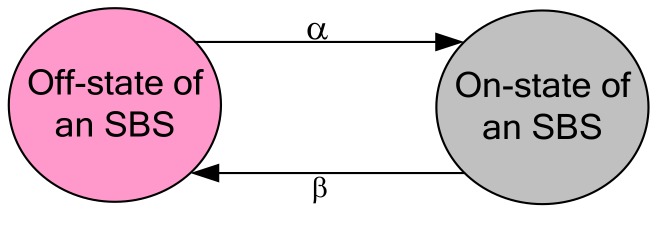
Two-state on/off transmission power model for an SBS.

According to [48], call arrivals or sessions can be modeled as a Poisson process. Since the transmission power of an SBS is switched on and off based on its UEs’ traffic requests, the transmission power of an SBS can be modeled as exponentially distributed continuous-time Poisson process such that the duration of any state of an SBS is exponentially distributed. Assume that each SBS has the maximum number of UEs of one. Since a UE would have either a traffic request or not at all at any time, a UE has only two states. Moreover, the switching of an SBS varies according to the variation in UE traffic requests such that an SBS also has two states. Since given the present state of an SBS, the future state is independent of the past such that the on-and off-state transitions of an SBS can be modeled as a two-state Markov chain as shown in Figure 6 where α denotes off-state to on-state transition rate, and β denotes on-state to off-state transition rate of an SBS. 

The dynamic switching of an SBS between on-and off-state can be captured by the average duration of each state, which is given by $\beta^{-1}$ and $\alpha^{-1}$ respectively for an on-state and an off-state [49]. Now, the probabilities that an SBS is at the on-and off-state can be given respectively as follows:

(1) $$\mathrm{Prob}\left(P_{\mathrm{on}-\mathrm{state}}^{\mathrm{tx}}\right)=\left(\beta^{-1}/\left(\alpha^{-1}+\beta^{-1}\right)\right)\;\Rightarrow \mathrm{Prob}\left(P_{\mathrm{on}-\mathrm{state}}^{\mathrm{tx}}\right)=\alpha /\left(\alpha +\beta \right)$$

Similarly:

(2) $$\mathrm{Prob}\left(P_{\mathrm{off}-\mathrm{state}}^{\mathrm{tx}}\right)=\left(\alpha^{-1}/\left(\alpha^{-1}+\beta^{-1}\right)\right)\;\Rightarrow \mathrm{Prob}\left(P_{\mathrm{off}-\mathrm{state}}^{\mathrm{tx}}\right)=\beta /\left(\alpha +\beta \right)$$

Hence, for a certain duration of time *T*, the average duration of an SBS at the on-and off-state can be determined according to its user service requirements, which is given respectively by:

(3) $$T_{\mathrm{on}-\mathrm{state}}=\left(\alpha /\left(\alpha +\beta \right)\right)\times T$$

(4) $$T_{\mathrm{off}-\mathrm{state}}=\left(\beta /\left(\alpha +\beta \right)\right)\times T$$

such that $T=T_{\mathrm{on}-\mathrm{state}}+T_{\mathrm{off}-\mathrm{state}}$.

However, if the switching is performed in times of TTIs, and the time-domain ABS-based eICIC is employed to an SBS, the above durations for an on-state and an off-state can be represented in terms of the number of TTIs defined by the time-domain resource scheduler. In general, in such cases, the value of *T*_on-state_ can be defined by whether or not the ABS-based eICIC technique, as well as the DTX power mechanism, are employed to an SBS as follows:

- If only the DTX power mechanism is applied to an SBS*, T*_on-state_ defines the total number of TTIs such that $T_{\mathrm{on}-\mathrm{state}}=\left|T_{\mathrm{on}-\mathrm{state}}^{\mathrm{with}\;\mathrm{DTX}}\right|=\left|T\backslash T_{\mathrm{off}-\mathrm{state}}^{\mathrm{with}\;\mathrm{DTX}}\right|$ over the evaluation period $\left|T\right|=Q$ TTIs during which an SBS serves its UE. $\left|T_{\mathrm{on}-\mathrm{state}}^{\mathrm{with}\;\mathrm{DTX}}\right|$ and $\left|T_{\mathrm{off}-\mathrm{state}}^{\mathrm{with}\;\mathrm{DTX}}\right|$ define respectively the number of TTIs during the on-state and the off-state with applying the DTX power mechanism to an SBS.
- However, if only the eICIC technique is applied to an SBS*, T*_on-state_ defines the total number of non-ABSs such that $T_{\mathrm{on}-\mathrm{state}}=\left|T_{\mathrm{non}-\mathrm{ABS}}^{\mathrm{without}\;\mathrm{DTX}}\right|=\left|T\backslash T_{\mathrm{ABS}}^{\mathrm{without}\;\mathrm{DTX}}\right|$ over the evaluation period $\left|T\right|=Q$ TTIs. $\left|T_{\mathrm{ABS}}^{\mathrm{wihout}\;\mathrm{DTX}}\right|$ and $\left|T_{\mathrm{non}-\mathrm{ABS}}^{\mathrm{without}\;\mathrm{DTX}}\right|$ define respectively the number of ABSs and non-ABSs without applying the DTX power mechanism to an SBS over the evaluation period $\left|T\right|=Q$ TTIs.
- Now, if both the ABS-based eICIC technique as well the DTX power mechanism are employed, *T*_on-state_ defines the actual number of non-ABSs such that $T_{\mathrm{on}-\mathrm{state}}=\left|T_{\mathrm{non}-\mathrm{ABS}}^{\mathrm{with}\;\mathrm{DTX}}\right|=\left|T_{\mathrm{non}-\mathrm{ABS}}^{\mathrm{without}\;\mathrm{DTX}}\backslash T_{\mathrm{non}-\mathrm{ABS}}^{\mathrm{no}\;\mathrm{traffic}}\right|$. $\left|T_{\mathrm{non}-\mathrm{ABS}}^{\mathrm{no}\;\mathrm{traffic}}\right|$ defines the number of non-ABSs with no traffic requests from any UE.
- Finally, if neither the ABS-based eICIC technique nor the DTX power mechanism is employed, then *T*_on-state_ defines the total number TTIs such that $T_{\mathrm{on}-\mathrm{state}}=\left|T\right|=Q$ over the evaluation period of *Q* TTIs during which an SBS serves its UE.

Note that the above expressions are valid for a single UE per SBS. For multiple UEs per SBS, the on-state and off-state probabilities can be found using the Birth-Death process [48], [50,51] as follows where *U*_max_ denotes the maximum number of UEs per SBS [52]:

(5) $$\mathrm{Prob}\left(P_{\mathrm{off}-\mathrm{state}}^{\mathrm{tx},\mathrm{mu}}\right)={1/\left(1+\alpha /\beta \right)}^{U_{\max}}$$

(6) $$\mathrm{Prob}\left(P_{\mathrm{on}-\mathrm{state}}^{\mathrm{tx},\mathrm{mu}}\right)=1-\left({1/\left(1+\alpha /\beta \right)}^{U_{\max}}\right)$$

### 3.2. Modeling 3D Clustering of In-Building Small Cell Base Stations

Referring to Figure 4, a 3D cluster of SBSs can be formed by allowing a minimum distance between SBSs located on the same floor as well as different floors of a multistory building subject to satisfying a minimum CCI level at an in-building UE, which is caused by one co-channel SBS to another due to reusing the same frequency to them. Assume that there is a uniform deployment of SBSs per building within a macrocell, and each apartment of any building has an SBS deployed in the middle of its ceiling. Then, following [43], the minimum distances in intra-floor $d_{\mathrm{intra}-\mathrm{floor}}{}^{*}$ and inter-floor $d_{\mathrm{inter}-\mathrm{floor}}{}^{*}$ levels can be expressed by (7) and (8) as follows where $\alpha_{\max,\mathrm{intra}-\mathrm{floor}}$ and $\alpha_{\max,\mathrm{inter}-\mathrm{floor}}$ represent the maximum allowable aggregate CCI in intra-floor and inter-floor levels respectively; $d_{\min}=5\;m$ represents a reference distance for the normalization of intra-and inter-floor interference; and $c_{\mathrm{intra}-\mathrm{floor}}$ and $c_{\mathrm{inter}-\mathrm{floor}}$ represent the maximum number of intra-floor and inter-floor co-channel SBSs, respectively:

(7) $$d_{\mathrm{intra}-\mathrm{floor}}^{*}\geq d_{\min}\;{\left(c_{\mathrm{intra}-\mathrm{floor}}/\alpha_{\max,\mathrm{intra}-\mathrm{floor}}\right)}^{3^{-1}}$$

(8) $$d_{\mathrm{inter}-\mathrm{floor}}^{*}\geq d_{\min}\;{\left(10^{-\;\left(\alpha_{\mathrm{floor}}\left(d_{\mathrm{inter}-\mathrm{floor}}^{*}\right)/10\right)}\;\left(c_{\mathrm{inter}-\mathrm{floor}}/\alpha_{\max,\mathrm{inter}-\mathrm{floor}}\right)\right)}^{\;3^{-1}}$$

A set of SBSs both at the intra-floor level $S_{\mathrm{intra}-\mathrm{floor}}$ and the inter-floor level $S_{\mathrm{inter}-\mathrm{floor}}$ subject to satisfying respectively the minimum distances of $d_{\mathrm{intra}-\mathrm{floor}}{}^{*}$ and $d_{\mathrm{inter}-\mathrm{floor}}{}^{*}$ can be defined. Note that $S_{\mathrm{inter}-\mathrm{floor}}$ is a multiplier representing the number of floors between co-channel SBSs at the inter-floor level. Hence, the total number of SBSs per 3D cluster in a multistory building can be found as follows:

(9) $$S_{F}=S_{\mathrm{intra}-\mathrm{floor}}\times S_{\mathrm{inter}-\mathrm{floor}}$$

Equation (9) signifies that due to exploiting the spatial-domain reuse of spectrum in a 3D building of SBSs, the same spectrum can be reused by a factor ζ, also termed as 3D spatial reuse factor, that can be expressed as follows:

(10) $$\zeta =S_{F,\mathrm{total}}/S_{F}$$

### 3.3. Modeling Secondary System for Dynamic Spectrum Sharing with In-Building Small Cells 

Recall that, we consider MNO 1 as the secondary system that uses the spectra of a number of primary systems to its in-building SBSs to enable each SBS with multiple spectrum bands to operate at multiple transceivers per SBS. The secondary system consists of a number of picocells deployed outdoor and a set of SBSs deployed in a number of multistory buildings within the coverage of a macrocell of MNO 1. The distribution of picocells and small cells in each building is assumed random and uniform. Denote *L* as the maximum number of buildings each deployed with an equal number of *S*_F,total_ SBSs for simplicity such that $s\in \left\{1,2,\ldots ,S_{F,\mathrm{total}}\right\}$._._ Recall that the number of small cell UEs per SBS is considered one. Further, assume that the number of macrocell UEs is denoted by *N* and a certain percentage *µ* of it is distributed randomly and non-uniformly within *L* buildings. Furthermore, we assume that whenever a macrocell UE is within the coverage of an SBS in a building, the corresponding UE is no longer served by the macrocell. Instead, the macrocell UE is offloaded to the SBS.

Both the macrocell and all picocells operate at the licensed 2-GHz microwave spectrum. However, for SBSs, we assume that each SBS is enabled with four transceivers as shown in Figure 3 to realize DSS techniques, namely CSA, CoPSA, LSA, and LAA. More specifically, transceiver 1 of each SBS operates at the same 2-GHz macrocell spectrum of its MNO 1 to realize CSA, transceiver 2 operates at the 28-GHz mmWave mobile system of MNO 2 to realize CoPSA by spectrum renting, transceiver 3 operates at the 3.5-GHz spectrum of a satellite system to realize LSA, and finally, transceiver 4 operates at the 60-GHz unlicensed mmWave spectrum of a WiFi system to realize LAA. Let *P*_SC,1_, *P*_SC,2_, *P*_SC,3_, and *P*_SC,4_ denote respectively the transmission powers and *M*_1_, *M*_2_, *M*_3_, and *M*_4_ denote respectively the number of resource blocks (RBs), corresponding to the operating spectrum bands 2-GHz of transceiver 1, 28-GHz of transceiver 2, 3.5-GHz of transceiver 3, and 60-GHz of transceiver 4 of each SBS.

### 3.4. Modeling Co-Channel Interference Management Technique 

As mentioned earlier, we consider ABS-based eICIC technique to avoid CCI due to sharing the same satellite spectrum with in-building SBSs. In the ABS-based eICIC technique as shown in Figure 2a, assume that ***T*** *=* {1, 2, 3, …, *Q*} represents the observation period or the simulation run time for the performance evaluation, and an APP consists of eight TTIs where a TTI is equal to 1 ms. Denote ***T*_ABS_** as the number of ABSs and ***T*_non-ABS_** as the number of non-ABSs such that ***T*_ABS_** *=* {*t: t =* 8*v+z*; *v =* 0, 1, 2,…,*Q*/8; *z =* 1,…,*T*_ABS_} where *T*_ABS_ *=* 1, 2,…,8 corresponds to ABS patterns φ *=*1/8, 2/8,…, 8/8 respectively and ***T*_non-ABS_** = ***T*\*T*_ABS_** resulting in ***T*** = ***T*_ABS_**+***T*_non-ABS_**. However, when no ABS-based eICIC technique is employed to SBSs, the observation period is given by ***T***. 

## 4. Problem Formulation

### 4.1. Performance Metrics for Small Cells in a Single Building 

A link throughput at RB = *i* in TTI = *t* in bps/Hz can be expressed as follows [51,53] where ε denotes the implementation loss factor:

(11) $$\sigma_{t,i}\left(\rho_{t,i}\right)=\left\{\begin{array}{ll} 0, & \rho_{t,i}<-10\;\mathrm{dB}\\ \varepsilon \log_{2}\left(1+10^{\left(\rho_{t,i}\left(\mathrm{dB}\right)/10\right)}\right)\;, & -10\;\mathrm{dB}\leq \rho_{t,i}\leq 22\;\mathrm{dB}\\ 4.4, & \rho_{t,i}>22\;\mathrm{dB} \end{array}\right\}$$

The aggregate capacity of all outdoor macrocell UEs of MNO 1 can be given by:

$$\sigma_{}^{\mathrm{otd}}=\sum_{t=1}^{Q}\sum_{i=1}^{M_{1}}\sigma_{t,i}\left(\rho_{t,i}\right)$$

where σ and ρ are responses over $M_{1}$ RBs of all outdoor macrocell UEs in *t*∈***T***. 

Recall that if an outdoor macrocell UE is present within the coverage of an in-building SBS of MNO 1, the macrocell UE is then served by the corresponding SBS causing no CCI with UEs of the SBS served by its transceiver 1. Further, in CoPSA by spectrum renting, since UEs of MNO 2 cannot get access to the rented portion of the 28-GHz mmWave spectrum of MNO 2, no CCI would occur between UEs of MNO 2 and any in-building SBS served by its transceiver 2. Likewise, due to operating at a different unlicensed 60-GHz spectrum from that of MNO 1, no CCI is generated with UEs of any in-building SBS served by its transceiver 4. Hence, there is no requirement for the application of CCI management technique to transceiver 1, transceiver 2, and transceiver 4 of any in-building SBS.

However, the presence of a satellite UE for LSA within the coverage of an SBS in a building generates CCI with UEs of the SBS served by its transceiver 3 due to reusing the same satellite spectrum to its transceiver 3, resulting in need of applying the ABS based eICIC technique to transceiver 3 of the SBS. Hence, to avoid CCI between a UE of an SBS served by transceiver 3 and a satellite UE, we consider that the satellite UE is served during ABSs, whereas a UE of the SBS is served during non-ABSs of each APP.

Due to considering an average performance estimation and relatively a less variation in channel characteristics such as the Doppler speed and the delay spread, particularly in high frequencies and in indoor environments, we consider an identical channel propagation characteristic for both microwave and mmWave spectra of each SBS per building for simplicity. Let Λ denote a set of transceivers per SBS such that $\Lambda \in \Lambda =\left\{1,2,3,4\right\}$. Hence, for a single building, i.e., *L* = 1, the aggregate capacity achieved by all four transceivers of an SBS *s* is given by:

(12) $$\sigma_{s,\Lambda \in \Lambda ,L=1}^{\mathrm{ind}}=\left\{\;\begin{array}{ll} \sum_{t\in T}\sum_{i=1}^{M_{1}}\sigma_{t,i}\left(\rho_{t,i}\right), & \mathrm{for}\;\mathrm{transceiver}\;1,\;i.e.,\;\Lambda =1\\ \sum_{t\in T}\sum_{i=1}^{M_{2}}\sigma_{t,i}\left(\rho_{t,i}\right), & \mathrm{for}\;\mathrm{transceiver}\;2,\;i.e.,\;\Lambda =2\\ \sum_{t\in T_{\mathrm{non}-\mathrm{ABS}}=T\backslash T_{\mathrm{ABS}}}\sum_{i=1}^{M_{3}}\sigma_{t,i}\left(\rho_{t,i}\right),\; & \mathrm{for}\;\mathrm{transceiver}\;3,\;i.e.,\;\Lambda =3\\ \sum_{t\in T}\sum_{i=1}^{M_{4}}\sigma_{t,i}\left(\rho_{t,i}\right), & \mathrm{for}\;\mathrm{transceiver}\;4,\;i.e.,\;\Lambda =4 \end{array}\right\}$$

where σ and ρ are capacity and SINR responses respectively over $M\in \left\{M_{1},M_{2},M_{3},M_{4}\right\}$ RBs corresponding to transceivers $\Lambda \in \Lambda =\left\{1,2,3,4\right\}$ of each SBS in time *t*. 

Then, for each 3D cluster of SBSs *S*_F_ per building, the average capacity corresponding to transceivers $\Lambda \in \Lambda =\left\{1,2,3,4\right\}$ is given by:

$$\sigma_{S_{F},\Lambda \in \Lambda ,L=1}^{\mathrm{ind}}=\left\{\begin{array}{cc} \sum_{s=1}^{S_{F}}\sum_{t\in T}\sum_{i=1}^{M_{1}}\sigma_{t,i}\left(\rho_{t,i}\right), & \mathrm{for}\;\Lambda =1\\ \sum_{s=1}^{S_{F}}\sum_{t\in T}\sum_{i=1}^{M_{2}}\sigma_{t,i}\left(\rho_{t,i}\right), & \mathrm{for}\;\Lambda =2\\ \sum_{s=1}^{S_{F}}\sum_{t\in T_{\mathrm{non}-\mathrm{ABS}}=T|T_{\mathrm{ABS}}}\sum_{i=1}^{M_{3}}\sigma_{t,i}\left(\rho_{t,i}\right), & \mathrm{for}\;\Lambda =3\\ \sum_{s=1}^{S_{F}}\sum_{t\in T}\sum_{i=1}^{M_{4}}\sigma_{t,i}\left(\rho_{t,i}\right), & \mathrm{for}\;\Lambda =4 \end{array}\right\}$$

So, the aggregate capacity served by a 3D cluster of *S*_F_ SBSs enabled with all transceivers $\Lambda \in \Lambda =\left\{1,2,3,4\right\}$ per building is given by:

(13) $$\sigma_{3D-\mathrm{cluster}}^{\mathrm{ind}}=\sum_{\Lambda =1}^{\Lambda =4}\sigma_{S_{F},\Lambda ,L=1}^{\mathrm{ind}}$$

Now, using (10), the overall aggregate capacity served by all SBSs *S*_F,total_ per building is given by:

(14) $$\sigma_{L=1}^{\mathrm{ind}}=\left(\zeta \times \sigma_{3D-\mathrm{cluster}}^{\mathrm{ind}}\right)$$

### 4.2. Ultra-Densification of Small Cells with L buildings

Assume that all buildings are built with the same type of materials and have an identical overall indoor structure and object effect. Hence, for a uniform distribution of UEs within the coverage of all SBSs for each of the *L* buildings, the indoor signal propagation characteristics do not deviate considerably from one building to another. In such cases, by linear approximation, the system-level average capacity of MNO 1 can be roughly given as follows:

(15) $$\sigma_{\mathrm{sys}}^{\mathrm{cap}}\left(L\right)=\sigma_{}^{\mathrm{otd}}+\left(L\times \sigma_{L=1}^{\mathrm{ind}}\right)$$

(16) $$\sigma_{\mathrm{sys}}^{\mathrm{cap}}\left(L\right)=\sum_{t=1}^{Q}\sum_{i=1}^{M_{1}}\sigma_{t,i}\left(\rho_{t,i}\right)+\left(L\times \left(\zeta \times \sum_{\Lambda =1}^{\Lambda =4}\sigma_{S_{F},\Lambda ,L=1}^{\mathrm{ind}}\right)\right)$$

Now, the average system-level SE of MNO 1 can be expressed as follows:

(17) $$\sigma_{\mathrm{sys}}^{\mathrm{SE}}\left(L\right)=\sigma_{\mathrm{sys}}^{\mathrm{cap}}\left(L\right)/\left(\left(M_{1}+M_{2}\right)\times Q\right)$$

Similarly, using the definition in Section 3.1, the average system-level EE without applying the DTX power mechanism to in-building SBSs is given by:

(18) $$\begin{array}{l} \sigma_{\mathrm{sys},\mathrm{without}\;\mathrm{DTX}}^{\mathrm{EE}}\left(L\right)=\\ \left(\begin{array}{l} \left(\begin{array}{l} \left(L\times \zeta \times S_{F}\right)\times \\ \left(P_{\mathrm{SC},1}+P_{\mathrm{SC},2}+\left(\left(\left|T_{\mathrm{non}-\mathrm{ABS}}^{\mathrm{without}\;\mathrm{DTX}}\right|/\left|T\right|\right)\times P_{\mathrm{SC},3}\right)+P_{\mathrm{SC},4}\right) \end{array}\right)\\ +\left(\left(S_{P}\times P_{\mathrm{PC}}\right)+\left(S_{M}\times P_{\mathrm{MC}}\right)\right) \end{array}\right)/\left(\sigma_{\mathrm{sys}}^{\mathrm{cap}}\left(L\right)/Q\right) \end{array}$$

However, the average system-level EE with applying the DTX power mechanism to in-building SBSs using the definition in Section 3.1 is given by:

(19) $$\begin{array}{l} \sigma_{\mathrm{sys},\mathrm{with}\;\mathrm{DTX}}^{\mathrm{EE}}\left(L\right)=\\ \left(\begin{array}{c} \left(\begin{array}{c} \left(L\times \zeta \times S_{F}\right)\times \\ \left(\begin{array}{c} \left(\left|T_{\mathrm{on}-\mathrm{state},\Lambda =1}^{\mathrm{with}\;\mathrm{DTX}}\right|/\left|T\right|\;\times P_{\mathrm{SC},1}\right)+\left(\left|T_{\mathrm{on}-\mathrm{state},\Lambda =2}^{\mathrm{with}\;\mathrm{DTX}}\right|/\left|T\right|\;\times P_{\mathrm{SC},2}\right)\\ +\left(\left(\left|T_{\mathrm{non}-\mathrm{ABS},\Lambda =3}^{\mathrm{without}\;\mathrm{DTX}}\right|/\left|T\right|\;\right)\times P_{\mathrm{SC},3}\right)+\left(\left|T_{\mathrm{on}-\mathrm{state},\Lambda =4}^{\mathrm{with}\;\mathrm{DTX}}\right|/\left|T\right|\;\times P_{\mathrm{SC},4}\right) \end{array}\right) \end{array}\right)\\ +\left(\left(S_{P}\times P_{\mathrm{PC}}\right)+\left(S_{M}\times P_{\mathrm{MC}}\right)\right) \end{array}\right)/\left(\sigma_{\mathrm{sys}}^{\mathrm{cap}}\left(L\right)/Q\right) \end{array}$$

**Remark** **1.** *In estimating system-level SE, we consider only the licensed spectrum of MNO 1 itself and the rented spectrum from MNO 2, since, unlike the licensed spectrum and the rented spectrum, no additional costs are needed to be paid by the MNO 1 due to operating at a shared, reused, or unlicensed spectrum by in-building SBSs. Since transceiver 3 of each in-building SBS of MNO 1 operates at the satellite spectrum under certain common understandings and negotiations with the satellite service provider, and transceiver 4 operates at the 60-GHz unlicensed spectrum, these spectra are not considered in estimating SE. However, transceiver 2 of each SBS operates at the licensed 28-GHz mmWave spectrum that is rented from MNO 2 by MNO 1, resulting in an additional cost paid by MNO 1 to MNO 2 in order to use the spectrum exclusively by its in-building SBSs. Due to this reason, the 28-GHz mmWave spectrum is considered in estimating SE. Note that, even though transceiver 1 operates at the reused spectrum of the macrocell of MNO 1 itself, this spectrum is considered in estimating SE. This is because we consider evaluating the system-level performance of MNO 1, not the performance of in-building SBSs only, which takes into account both the licensed macrocell spectrum as well as the spectrum of in-building SBSs of MNO 1 as given by (17).*

**Remark** **2.** *Note that the minimal optimal conditions for EE and SE are not the same for all transceivers since different transceivers operate at different frequency bands. In general, due to experiencing more penetration loss through internal walls and floors of a multistory building by a mmWave signal than a microwave signal, transceivers operating at a mmWave have relatively smaller 3D cluster size than those operating at a microwave spectrum, resulting in allowing more reuse of a mmWave spectrum and hence gaining higher SE and EE than its counterpart microwave spectra. However, there are mainly two approaches to consider a common 3D cluster size for all transceivers operating at different frequencies as follows:*

*1)*  *Since a microwave spectrum requires a larger 3D cluster size than a mmWave spectrum, the simplest approach is to consider the minimum distance between co-channel SBSs based on the lowest microwave spectrum (i.e., 2-GHz) such that the CCI thresholds for both mmWave and microwave spectra can be satisfied.*
*2)*  *The second approach is to relax or increase the interference thresholds of microwave spectra to decrease the cluster size as compared to the mmWave spectra such that the cluster sizes due to operating microwave spectra and mmWave spectra would become the same.*

**Remark** **3.** *Recall that the multi-cell interference among small cells and macrocells due to reusing spectra within in-building small cells is addressed by exploiting space-domain, as well as time-domain. More specifically, exploiting space-domain, a 3D cluster of SBSs is formed subject to satisfying the co-channel interference threshold to avoid multi-cell interference for SBSs within a building. Further, to avoid co-channel interference among SBSs per 3D cluster, as well as per building, we consider the multi-cell co-channel interference management by exploiting time-domain using the ABS based eICIC technique such that spectra can be allocated orthogonally in both time-and frequency-domain to SBSs. Furthermore, an indoor macrocell UE is assumed to be offloaded to an SBS whenever it is found within a building such that no co-channel interference can occur among macrocell UEs and small cell UEs even though transceiver 1 of each SBS operates at the same macrocell spectrum.*

### 4.3. Optimality in EE and SE Performances

From (16) and (17), it can be found that capacity and hence SE is a linearly increasing function of *L*, whereas (18) and (19) indicate that the value of EE is relatively high when *L* is small and is low when *L* is large, resulting in EE a decreasing function of *L.* Hence, at a certain value of *L* = L^*^, EE and SE curves must intersect each other. Since any value of *L* greater than L^*^ causes improvement in both SE and EE performances, an optimal value of EE, as well as SE, can be defined by considering a value of *L* higher than the value of L=L^*^ at the point of intersection and can be derived as follows:

$$\mathrm{at}\;L=L^{*},\;\sigma_{\mathrm{sys}}^{\mathrm{SE}}\left(L\right)=\sigma_{\mathrm{sys},\mathrm{with}\;\mathrm{DTX}}^{\mathrm{EE}}\left(L\right)$$

such that using (17) and (19), we can find the following:

$$\begin{array}{l} \Rightarrow \sigma_{\mathrm{sys}}^{\mathrm{cap}}\left(L\right)/\left(\left(M_{1}+M_{2}\right)\times Q\right)=\left(\left(\left(L\times \zeta \times S_{F}\right)\times P_{\mathrm{TX},S}\right)+P_{\mathrm{TX},M}\right)/\left(\sigma_{\mathrm{sys}}^{\mathrm{cap}}\left(L\right)/Q\right)\\ \Rightarrow \sigma_{\mathrm{sys}}^{\mathrm{cap}}{\left(L\right)}^{2}/\left(\left(M_{1}+M_{2}\right)\times Q^{2}\right)=\left(\left(L\times \zeta \times S_{F}\right)\times P_{\mathrm{TX},S}\right)+P_{\mathrm{TX},M}\\ \Rightarrow {\left(\sigma_{\mathrm{sys}}^{\mathrm{cap}}\left(L\right)/Q\right)}^{2}/\left(M_{1}+M_{2}\right)=\left(\left(L\times \zeta \times S_{F}\right)\times P_{\mathrm{TX},S}\right)+P_{\mathrm{TX},M}\\ \Rightarrow \left({\left(\sigma_{\mathrm{sys}}^{\mathrm{cap}}\left(L\right)/Q\right)}^{2}/\left(M_{1}+M_{2}\right)\right)-P_{\mathrm{TX},M}=\left(\left(L\times \zeta \times S_{F}\right)\times P_{\mathrm{TX},S}\right) \end{array}$$

Hence, an optimal value of *L* = L^*^ can be expressed as:

(20) $$\begin{array}{l} \Rightarrow L^{*}=\frac{\left(\left({\left(\sigma_{\mathrm{sys}}^{\mathrm{cap}}\left(L\right)/Q\right)}^{2}/\left(M_{1}+M_{2}\right)\right)-P_{\mathrm{TX},M}\right)}{\left(\zeta \times S_{F}\times P_{\mathrm{TX},S}\right)}\\ \Rightarrow L^{*}=\frac{\left(\left({\left(\begin{array}{c} \left(\sum_{t=1}^{Q}\sum_{i=1}^{M_{1}}\sigma_{t,i}\left(\rho_{t,i}\right)\right) \end{array}/Q\right)}^{2}/\left(\begin{array}{c} M_{1}+\\ M_{2} \end{array}\right)\right)-\left(\begin{array}{c} \left(S_{P}\times P_{\mathrm{PC}}\right)+\\ \left(S_{M}\times P_{\mathrm{MC}}\right) \end{array}\right)\right)}{\left(\begin{array}{c} \left(\zeta \times S_{F}\times \left(\begin{array}{c} \left(\left|T_{\mathrm{on}-\mathrm{state},\Lambda =1}^{\mathrm{with}\;\mathrm{DTX}}\right|/\left|T\right|\;\times P_{\mathrm{SC},1}\right)+\left(\left|T_{\mathrm{on}-\mathrm{state},\Lambda =2}^{\mathrm{with}\;\mathrm{DTX}}\right|/\left|T\right|\times P_{\mathrm{SC},2}\right)\\ +\left(\left(\left|T_{\mathrm{non}-\mathrm{ABS},\Lambda =3}^{\mathrm{with}\;\mathrm{DTX}}\right|/\left|T\right|\right)\times P_{\mathrm{SC},3}\right)+\left(\left|T_{\mathrm{on}-\mathrm{state},\Lambda =4}^{\mathrm{with}\;\mathrm{DTX}}\right|/\left|T\right|\times P_{\mathrm{SC},4}\right) \end{array}\right)\right)\\ -\left({\left(\begin{array}{c} \left(\zeta \times \sum_{\Lambda =1}^{\Lambda =4}\sigma_{S_{F},\Lambda ,L=1}^{\mathrm{ind}}\right) \end{array}/Q\;\right)}^{2}/\left(\begin{array}{c} M_{1}+\\ M_{2} \end{array}\right)\right) \end{array}\right)} \end{array}$$

Using (20), an optimal value of *EE* can be expressed as:

(21) $$\begin{array}{l} \Rightarrow \sigma_{\mathrm{sys},\mathrm{with}\;\mathrm{DTX}}^{\mathrm{EE}}{\left(L^{*}\right)}^{*}=\\ \left(\begin{array}{c} \left(\begin{array}{c} \left(L^{*}\times \zeta \times S_{F}\right)\\ \times \left(\begin{array}{c} \left(\left|T_{\mathrm{on}-\mathrm{state},\Lambda =1}^{\mathrm{with}\;\mathrm{DTX}}\right|/\left|T\right|\;\times P_{\mathrm{SC},1}\right)\\ +\left(\left|T_{\mathrm{on}-\mathrm{state},\Lambda =2}^{\mathrm{with}\;\mathrm{DTX}}\right|/\left|T\right|\;\times P_{\mathrm{SC},2}\right)\\ +\left(\left(\left|T_{\mathrm{non}-\mathrm{ABS},\Lambda =3}^{\mathrm{with}\;\mathrm{DTX}}\right|/\left|T\right|\;\right)\times P_{\mathrm{SC},3}\right)\\ +\left(\left|T_{\mathrm{on}-\mathrm{state},\Lambda =4}^{\mathrm{with}\;\mathrm{DTX}}\right|/\left|T\right|\;\times P_{\mathrm{SC},4}\right) \end{array}\right) \end{array}\right)\\ +\left(\left(S_{P}\times P_{\mathrm{PC}}\right)+\left(S_{M}\times P_{\mathrm{MC}}\right)\right) \end{array}\right)/\left(\left(\begin{array}{c} \sum_{t=1}^{Q}\sum_{i=1}^{M_{1}}\sigma_{t,i}\left(\rho_{t,i}\right)\\ +\left(\begin{array}{c} L^{*}\times \zeta \times \\ \sum_{\Lambda =1}^{\Lambda =4}\sigma_{S_{F},\Lambda ,L=1}^{\mathrm{ind}} \end{array}\right) \end{array}\right)/Q\;\right)\; \end{array}$$

Similarly, an optimal value of *SE* can be expressed as:

(22) $$\Rightarrow \sigma_{\mathrm{sys}}^{\mathrm{SE}}{\left(L^{*}\right)}^{*}=\left(\begin{array}{l} \sum_{t=1}^{Q}\sum_{i=1}^{M_{1}}\sigma_{t,i}\left(\rho_{t,i}\right)+\\ \left(L^{*}\times \zeta \times \sum_{\Lambda =1}^{\Lambda =4}\sigma_{S_{F},\Lambda ,L=1}^{\mathrm{ind}}\right) \end{array}\right)/\left(\left(M_{1}+M_{2}\right)\times Q\right)$$

### 4.4. Algorithm for the Proposed Framework

#### 4.4.1. Principle of Operation of the Algorithm 

Algorithm 1 presents the logical operation of the proposed framework, exploiting enabling techniques in time, frequency, power, and space domains to maximize capacity, SE and EE of a mobile system using in-building small cells. The algorithm works as follows. We consider arbitrary values of on-state and off-state durations of an SBS based on whether or not the ABS based eICIC technique, as well as the DTX power mechanism, are employed to the SBSs. Further, for certain values of $\alpha_{\max,\mathrm{intra}-\mathrm{floor}}$ and $\alpha_{\max,\mathrm{inter}-\mathrm{floor}}$, using (7)–(10), the size of a 3D cluster of SBSs for each building is estimated to find ζ per building. The ABS based eICIC technique is employed only to the third transceiver of each SBS operating simultaneously with a satellite system by reusing its spectrum. The capacity achieved per transceiver per SBS is estimated first, followed by aggregating the capacities achieved from all the transceivers per SBS using (12). Likewise, the aggregate capacity per 3D cluster of SBSs using (13) and then per building using (14) is estimated. The system-level capacity, SE and, EE performances with, as well as without, employing the DTX power mechanism for more than one building of SBSs, i.e., *L*>1 are then estimated by increasing the value of *L.* Finally, based on the EE and SE responses, the value of a minimum optimal point in terms of *L* corresponding to the intersection of the normalized EE and SE is found to define an optimal region for SE and EE performances for a cost-efficient deployment of in-building SBSs.
**Algorithm 1.** A proposed framework to maximize SE and EE using in-building small cells.
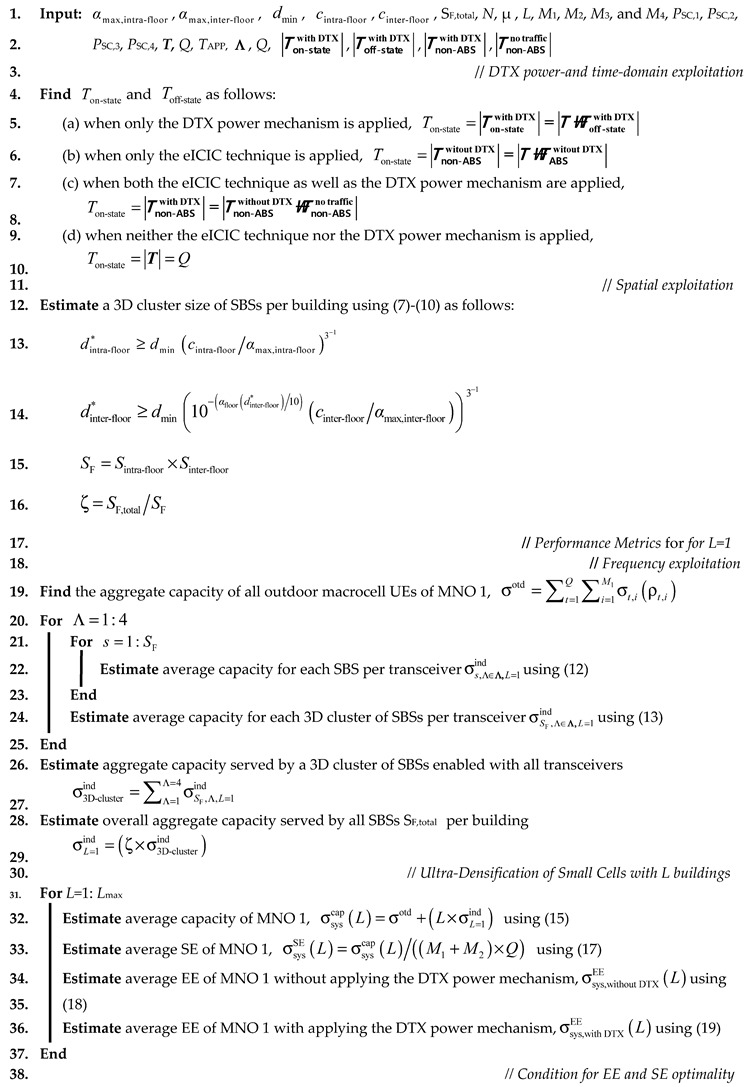

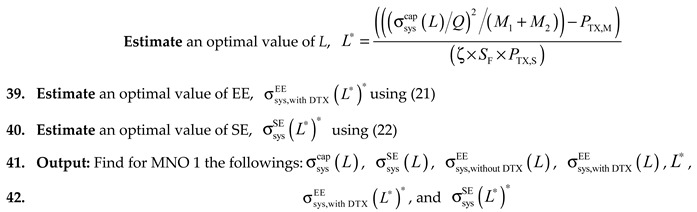


#### 4.4.2. Computational Complexity and Optimality Analysis of Algorithm 1

We consider carrying out the computational time complexity of Algorithm 1. In computational complexity function, higher-order terms dominate lower ones for large input sizes, resulting in considering only the higher ones is sufficient enough. Following so, since in Algorithm 1, lines 19 to 34 mainly dominate the overall computational time of Algorithm 1, considering only this section, ranging between lines 19 and 34, is sufficient to compute the runtime of Algorithm 1. Note that the variables that affect substantially the run-time of Algorithm 1 are *L*, ζ, $S_{F}$, Q*,*$M_{1},M_{2},M_{3},\;\mathrm{and}\;M_{4}$. Assume that $M_{1}=M_{2}=M_{3}=M_{4}=M$, which results in the total system bandwidth in terms of the number of resource blocks $M_{T}=M_{1}+M_{2}+M_{3}+M_{4}=4M$. Note that, the values of ζ, $S_{F}$, Q, and M are finite integers such that their multiplication also becomes a finite constant. In this regard, if we assume that *f* denotes a function representing the run-time of Algorithm 1 for these inputs, then it can be found that $f\left(L,\zeta ,S_{F},Q,M\right)≅O\left(L\right)$. Hence, the run-time of Algorithm 1 grows linearly with *L*. Since the maximum number of multistory buildings *L* that can be built over the coverage of a macrocell is a finite positive integer, i.e., 1 < *L* << ∞, the run-time of Algorithm 1 $O\left(L\right)$ is also finite for any large input size of *L* limited by the coverage area of a macrocell. Since Algorithm 1 requires the run-time $O\left(L\right)$, i.e., upper bounded, Algorithm 1 is asymptotically optimal [54]. 

## 5. Default Estimation, Parameter, and Assumption 

### 5.1. Estimation of Indoor 28-GHz Millimeter-Wave Path Loss 

In indoor environments, the large-scale shadowing effect increases with an increase in mmWave frequency due to increasing diffuse scattering and diffraction loss, whereas decreasing reflection at smaller wavelength, resulting in frequency dependency and higher path loss in indoor channels than that in outdoor channels [55]. Moreover, even though directional path loss models for mmWave are suitable [56,57,58,59,60,61], standards bodies recommend omnidirectional path loss models due to considering arbitrary antenna patterns [55]. Furthermore, according to [62], the small-scale fading effect is not influential on the multi-path components of received signals in mmWave frequencies (e.g.,±3 dB fading variation between the maximum and the minimum received power level). 

Hence, to incorporate the frequency-dependency and omnidirectional antenna radiation pattern features into the path loss with insignificant small-scale fading effect for mmWave signals in indoor environments, we consider 28-GHz omnidirectional multi-frequency combined polarization *close-in free space reference distance with frequency-dependent path loss exponent* (CIF) large-scale path-loss model for transceiver 2 of each SBS. CIF employs a physically-based reference distance *d*_0_ =1 m for stability and is given by:

$$PL[\mathrm{dB}]=10\log_{10}{\left(4\Pi d_{0}f_{c}/c\right)}^{2}+10n\left(1+b\left(f-f_{0}/f_{0}\right)\right)\log_{10}\left(d/d_{0}\right)+X_{\Delta }$$

Hence, for 28-GHz mmWave signals, the above equation for CIF can be expressed as follows:

(23) $$PL[\mathrm{dB}]=61.38+10n\left(1+b\left(f-f_{0}/f_{0}\right)\right)\log_{10}\left(d/d_{0}\right)+X_{\Delta }$$

where *n* denotes path loss exponent, *b* represents the slope of linear frequency dependence of path loss, *f*_0_ represents a fixed reference frequency serving as the balancing point of the linear frequency dependence of *n* and $X_{\Delta }$ is the Gaussian random variable with standard deviation Δ representing large-scale signal variation about the mean path loss due to shadowing. Table 1 shows the values of these parameters for both LOS and NLOS environments.

### 5.2. Estimation of 3D Cluster Size and Spatial Reuse of Spectrum per Building 

Using Figure 4, considering only the first-tier of co-channel interferers, the maximum aggregate intra-floor CCI that a UE within the coverage of its serving SBS experiences when the value of $c_{\mathrm{intra}-\mathrm{floor}}$ is the maximum of 8 irrespective of the number of apartments per floor. Likewise, the maximum aggregate intra-floor CCI occurs when the value of $c_{\mathrm{inter}-\mathrm{floor}}$ is the maximum of 2. In other words, the maximum number of co-channel interferers for a UE is 10, 8 in intra-floor level and 2 in inter-floor level, for the worst-case CCI effect. Assume that the maximum allowable aggregate interference effect in the intra-floor level and inter-floor level for a UE is limited by $\alpha_{\max,\mathrm{intra}-\mathrm{floor}}=0.57$ and $\alpha_{\max,\mathrm{inter}-\mathrm{floor}}=0.044$ respectively in normalized value where normalization is performed with respect to the maximum value of per link CCI at a reference distance, $d_{\min}=5\;m$.

Now, from (7) and (8), using these assumed values, the minimum co-channel interferer distances in intra-floor and inter-floor levels are given by $d_{\mathrm{intra}-\mathrm{floor}}{}^{*}\geq 12\;m$ and $d_{\mathrm{inter}-\mathrm{floor}}{}^{*}\geq 7.07\;m$ respectively. However, using Figure 4, the vertical distance from an inter-floor co-channel interferer is given by, $d_{\mathrm{ver}}{}^{*}\geq 5\;m$ where $d_{\mathrm{ver}}=\sqrt{\left(d_{\mathrm{inter}-\mathrm{floor}}{}^{2}-d_{\min}{}^{2}\right)}$. Now, to satisfy a minimum distance between adjacent co-channel interferers of $d_{\mathrm{intra}-\mathrm{floor}}{}^{*}\geq 12\;m$ in intra-floor and $d_{\mathrm{ver}}{}^{*}\geq 5\;m$ in inter-floor level, the frequency reuse can be performed at least one adjacent apartment apart intra-floor level and one floor apart in inter-floor level (Figure 4). Note that the side length of a square apartment is 10 m and the distance between adjacent floors is 3 m. Hence, considering these minimum distances, using (9), a 3D cluster of SBSs consists of a total of *S*_F_ = 8 SBSs in 2 adjacent floors, each having 4 SBSs as shown in Figure 4. Now, using (10), the same frequency can be reused by a factor ζ=64/8=8. Similarly, following the above procedure, *S*_F_ and ζ can be estimated for any arbitrary values of $c_{\mathrm{intra}-\mathrm{floor}}$ and $c_{\mathrm{inter}-\mathrm{floor}}$ set by an MNO, for example.

### 5.3. Default Parameters and Assumptions

Table 2 shows the default parameters and assumptions used for the system-level performance evaluation. Due to a small coverage area and a low transmission power of an SBS, the similar signal propagation characteristics of all SBSs located within the same building or different buildings are assumed for simplicity in modeling. Other than 60-GHz, all spectrum bands, including 2-GHz, 3.5-GHz, and 28-GHz, are considered as licensed spectrum bands. Due to high-frequency bands used in indoor environments, which help reduce the multipath fading effect, we consider line-of-sight (LOS) models with large-scale shadowing effects for 28-GHz and 60-GHz mmWave bands.

However, for 2-GHz and 3.5-GHz bands, both small-scale fading and large-scale fading effects are considered. Channel models for all spectrum bands are considered as recommended by 3GPP [63], ITU-R [64] and empirical measurement-based studies [55].

We consider an aggregate value of CCI threshold as a constraint at a serving small cell UE due to the presence of co-channel SBSs in both intra-floor and inter-floor levels to reuse the same spectrum more than once within each building. Further, to estimate a common size of a 3D cluster of SBSs in each building for all operating spectra per SBS, we consider setting a minimum distance between co-channel SBSs based on the lowest microwave spectrum (i.e., 2-GHz) such that the same value of CCI thresholds for both 28-GHz and 60-GHz mmWave spectra, as well as both 2-GHz and 3.5-GHz microwave spectra, can be chosen. 

In modeling the DTX power mechanism, we assume that the switching delay due to transitioning between on-state and off-state, as well as the processing delay due to processing user traffic requests, are negligible. Moreover, in practice, although the on-state and off-state durations for each SBS varies according to its UE traffic demand, for simplicity, the on-state and off-state durations are determined for one SBS based on the requirements of the SBS over the simulation period *T* for each performance evaluation scenario. The ABS-based eICIC technique is employed only to the 3.5-GHz band to avoid CCI between UEs of any SBS and the satellite system. Moreover, due to an optimal trade-off between fairness and throughput performances, we consider Proportional Fair frequency-domain schedulers for all in-building SBSs. Note that, unless declared explicitly, parameters and assumptions used in this paper for the performance evaluation are taken in Table 2.

## 6. Performance Results

### 6.1. Frequency Exploitation

**Figure 7:**
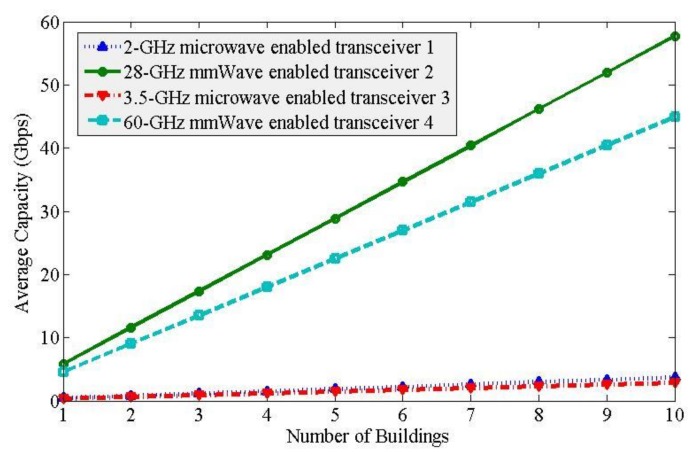
Average capacity responses of multiple spectrum bands per SBS with the variation of *L*.

Figure 7 shows the average capacity responses of all transceivers per SBS with the variation of the number of buildings. From Figure 7, for mmWave spectra, it can be found that the 28-GHz LOS mmWave spectrum gives better capacity performance than that of 60-GHz LOS mmWave. This is because the path loss in indoor environments is frequency-dependent and increases with an increase in frequency. Whereas, for microwave spectra, due to the NLOS small-scale multipath fading effect, the microwave spectra operating at transceivers 1 and 3 provide lower capacity performance than mmWave spectra operating at transceiver 2 and 4. Moreover, due to enforcing the ABS based eICIC technique to transceiver 3, the capacity achieved from the satellite spectrum is less than that of the MNO 1 spectrum for the same amount of spectrum at 2-GHz and 3.5-GHz.

Hence, to address the enormous capacity demand for future 5G mobile networks, microwave spectra are not sufficient even with applying DSS due to their inherent poor signal propagation characteristics. In addition to microwave spectra, mmWave spectra with a large amount of available spectrum, as well as due to their favorable channel characteristics within a short distance in indoor environments, can address the expected bulk amount of indoor user data at a high data rate for 5G mobile systems. In short, both the quality in terms of spectrum characteristics as well as the quantity in terms of the number of spectrum bands are needed to exploited to address high indoor capacity demand. This implies that, rather than using the traditional single-band enabled SBSs, SBSs enabled with multiple spectrum bands in both microwave as well as mmWave ranges as shown in Figure 7 such that the capacity delivered by each SBS is equal to the summation of capacities achieved by each of the multiple bands can address the expected high indoor capacity demand of future 5G mobile systems.

### 6.2. DTX Power Exploitation

**Figure 8:**
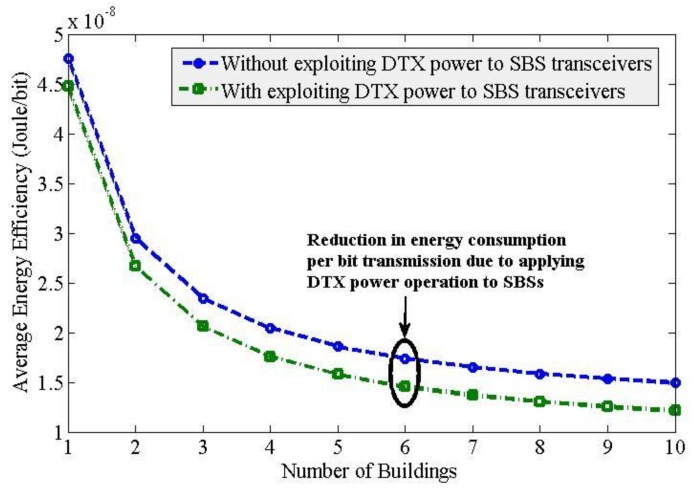
Average system-level EE responses with applying the DTX power mechanism for an average on-state duration of 75% to each transceiver of each SBS for all buildings.

**Figure 9:**
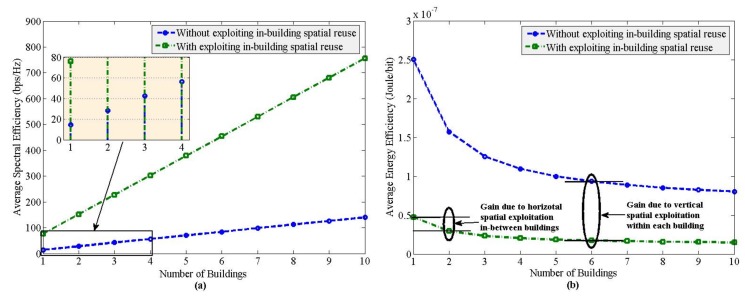
Average system-level (**a**) SE and (**b**) EE responses due to the spatial reuse of the same spectrum both vertically and horizontally to in-building SBSs without applying the DTX power mechanism for ζ = 6.

To evaluate the performance using the DTX power mechanism, we assume that each transceiver serves only 75% of the total time *T* to achieve the respective capacity shown in Figure 7. Following so, Figure 8 shows the average EE performance of SBSs when the DTX power mode is applied for an average on-state duration of 75% and an off-state duration of 25% to each SBS. Since the transmission power of an SBS is reduced by 25% for each transceiver, the energy consumed per bit transmission is reduced by 25% as well irrespective of the spatial reuse of spectrum both horizontally and vertically. This results in an SBS of 25% more efficient when it is enabled with the DTX power mechanism than that without enabling it with the DTX power mechanism as shown by the EE gap in Figure 8. It is to be noted that, due to applying the ABS based eICIC technique to transceiver 3 of each SBS, we assume that the DTX power mechanism is applied during non-ABSs such that the non-traffic duration during non-ABSs is considered as 25%. More specifically, $\left|T_{\mathrm{non}-\mathrm{ABS}}^{\mathrm{no}\;\mathrm{traffic}}\right|$= 0.25 such that $\left|T_{\mathrm{non}-\mathrm{ABS}}^{\mathrm{with}\;\mathrm{DTX}}\right|=\left|T_{\mathrm{non}-\mathrm{ABS}}^{\mathrm{without}\;\mathrm{DTX}}\backslash T_{\mathrm{non}-\mathrm{ABS}}^{\mathrm{no traffic}}\right|$ = 0.75. Further, since the same capacity is achieved by each transceiver of each SBS with applying the DTX power mechanism, there is no change in average SE responses with applying the DTX power mechanism from that without applying the DTX power mechanism to SBSs as shown in Figure 9. 

### 6.3. Spatial Exploitation 

To evaluate spatial exploitation, vertical spatial reuse of spectrum (i.e., ζ) can be exploited using (7)-(10) by forming 3D clusters of SBSs within each building. Whereas by changing the number of buildings of SBSs *L*, horizontal spatial reuse of spectrum in each building of SBSs can be exploited. Figure 9 shows exploiting both the vertical and horizontal spatial reuse of the same spectrum without applying the DTX power mechanism to in-building SBSs. From Figure 9, it can be found for horizontal densification of SBSs that, even though SE increases proportionally with an increase in *L*, EE gets steady for large values of *L* for both orthogonal spectrum sharing (OSS) and non-orthogonal spectrum sharing (nOSS) for ζ = 6. Further, nOSS provides higher SE and EE performances than OSS irrespective of the value of *L*. 

**Figure 10:**
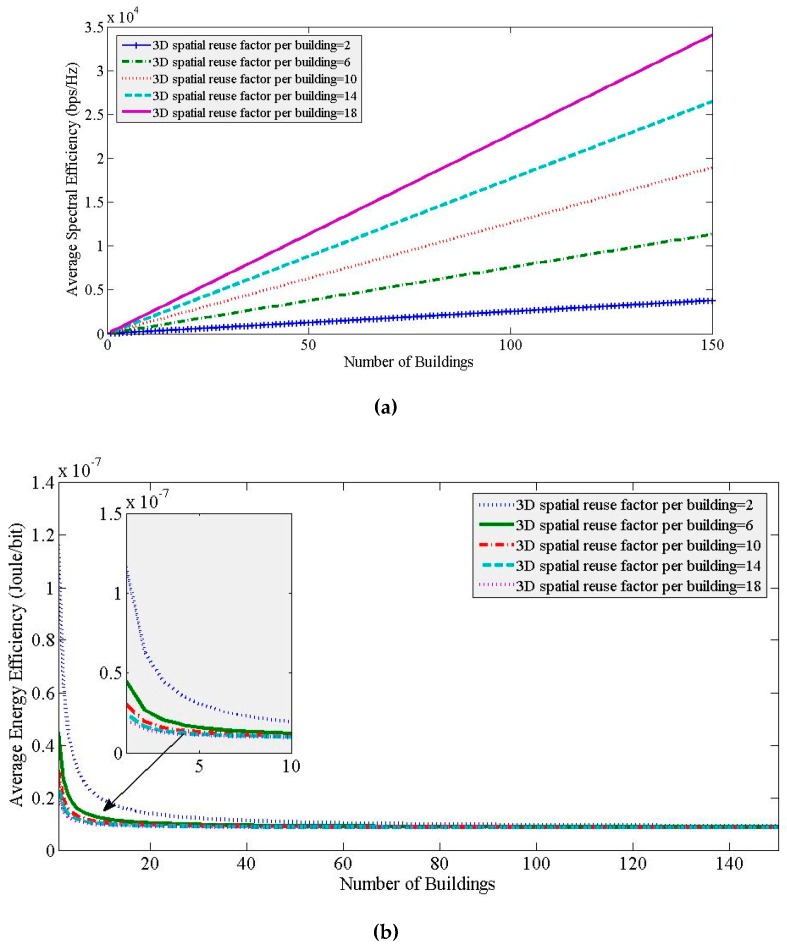
SE and EE responses with the variation of 3D spatial reuse factor, hence the number of 3D clusters of SBSs, within a building. (**a**) average SE versus the number of buildings of SBSs, (**b**) average EE versus the number of buildings of SBSs.

If we now vary the 3D spatial reuse factor ζ per building to show the impact of vertical densification by changing the aggregate interference threshold, it can be found that the SE increases proportionately for any value of *L*, whereas the EE does not increase considerably except for low values of *L* with an increase in ζ as shown in Figure 10. This implies that vertical densification shows similar SE and EE trends in performances with *L* irrespective of the value of ζ. Since spatial reuse of spectrum, both vertically and horizontally, to SBSs per building improves SE and EE performances, depending on the environmental and building profiles of a certain area, either horizontal or vertical, or both spatial reuses of the spectrum can be exploited to achieve both SE and EE targets. Since nOSS explores both horizontal and vertical spatial reuse of spectrum, nOSS is more scalable than OSS.

### 6.4. Condition for Optimality of a Cost-Efficient Small Cell Deployment

**Figure 11:**
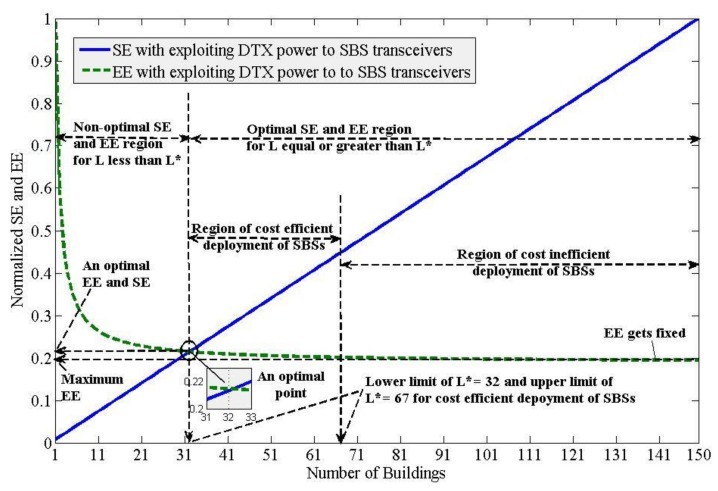
Optimal values of SBS density, SE, and EE for a cost-efficient deployment of SBSs for ζ = 6.

Figure 11 shows normalized SE and EE responses by varying only the horizontal densification of SBSs (i.e., *L*) for ζ = 6 and exploiting power, frequency, time and space-domain to reuse spectra to in-building SBSs. Using (20), the minimum optimal value of *L* can be found in Figure 11, which is approximately equal to 32. This minimum optimal value of *L* can be justified by the fact that any value of *L*, which is less than 32, results in decreasing both SE and EE (i.e., consuming more energy per bit transmission to obtain less aggregate capacity per unit frequency). However, any value of *L*, which is equal or greater than 32, results in increasing both SE and EE such that more aggregate capacity per unit frequency can be obtained at the cost of less energy required per bit transmission of SBSs. 

Recall that, even though SE improves linearly with an increase in the density of SBSs horizontally (i.e., *L*), EE improves incrementally and gets almost constant for some values of *L*, for example, *L* = 67 in Figure 11 such that any value of *L* greater than 67 results in no improvement in EE. Hence, this, in turn, causes an inefficient deployment of SBSs horizontally since an increase in *L* (i.e., the density of SBSs horizontally) causes to increase both deployment and operational costs of SBSs. This implies that, an upper limit of *L* due to which both SE and EE improve can be given by, *L* = *L*_max_ = 67. Hence, using these values of *L*, we can now define an optimal region for a cost-efficient deployment of SBSs, which is given by 32 ≤ *L* ≤ 67. 

Therefore, for a given vertical spatial-reuse pattern of the spectrum, a cost-efficient deployment of SBSs that can result in both EE and SE improvement can be found by choosing a value of *L* that falls within the range for optimality of *L*. In short, there is an upper limit to the horizontal densification of SBSs for a cost-efficient deployment. Now using (21) and (22), the minimum optimal normalized value for both SE and EE can be found from Figure 11 is 0.22, which respectively defines in absolute values of 0.22 × EE_min_ = $\left(0.22\times 0.44842\times 10^{-7}\right)$= 9.864 nJ/b for EE and 0.22×SE_max_=$0.22\times 1.1342\times 10^{4}$ Mbps/Hz = 2495.2 Mbps/Hz for SE. Since EE does not change for *L* ≥ *L*_max_, using Figure 11, the maximum value of EE is given by, EE_max_ = 0.2×EE_min_= $\left(0.2\times 0.44842\times 10^{-7}\right)$ = 8.968 nJ/b.

**Figure 12:**
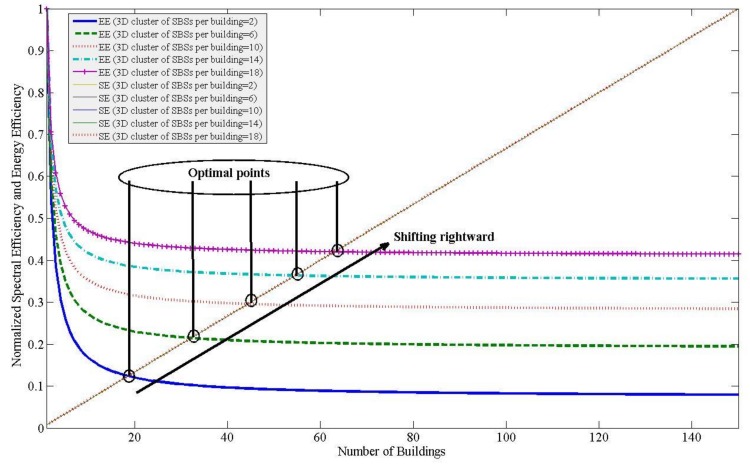
SE and EE responses and the condition for optimality due to varying both ζ and *L*.

Now, if we vary the densification of SBSs both horizontally and vertically (i.e.,ζ), the normalized responses of SE and EE can be found as shown in Figure 12. From Figure 12, it can be found that the minimum optimal point (i.e., the region for optimality) shifts rightward with an increaseζ due to improving EE responses, resulting in an increase in *L* as well. Hence, the point of optimality is affected by both horizontal and vertical spectrum reuse to justify a cost-efficient deployment of SBSs. Note that the normalized values for the SE do not change with a change in ζ since the SE is a function of the average capacity of SBSs only, which is scaled by the maximum value of SE for any value of ζ. Whereas the EE response varies with ζ since the EE is a function of both the transmission power as well as the average capacity of SBSs.

### 6.5. Summary 

In summary, both EE and SE can be improved by exploiting the major four domains, namely frequency, time, power, and space. More specifically, firstly, by exploiting frequency-domain, as compared to a single-band, multiband enabled SBSs can achieve higher SE and EE. In this case, to acquire multiple bands, DSS techniques can be applied such that the spectrum of other homogenous systems (e.g., another mobile system), as well as heterogeneous systems (e.g., a satellite system), can be shared with in-building small cells. 

Secondly, by exploiting spatial-domain, the same set of spectra can be reused to each horizontally separated multiband enabled SBS on 2D space of earth. Moreover, due to the floor and internal wall penetration losses, vertically separated SBSs located on different floors of a building can be grouped under certain constraints to form 3D clusters of SBSs both intra-floor and inter-floor levels such that the whole spectrum of any band can be reused to SBSs of each 3D cluster. Hence, by considering both horizontal and vertical spatial exploitation, the same set of spectra can be reused to both horizontally and vertically separated multiband enabled SBSs more than once to improve both SE and EE performances as shown in Figure 9. 

Thirdly, since the user-generated traffic is not always available, the transmission power of a multi-band enabled SBS can be made adaptive such that an SBS can go to an off-state when no traffic requests are available within its coverage. However, when a user data traffic request arrives, an SBS turns into its on-state from its off-state to serve the user-generated traffic. Hence, instead of keeping the transmission power of an SBS always on, the SBS can be turned on and off based on the traffic demand, which in literature termed as the DTX power operation. Due to reducing the consumption of energy per bit transmission, the EE response improves significantly as shown in Figure 8. 

Fourthly, to operate multiple spectra using DSS techniques at any multiband enabled SBS from either the same or different systems, it is necessary to avoid CCI originated due to sharing the same spectrum of another system with the SBS. To address this issue, we consider the time-domain ABS-based eICIC technique such that UEs of an SBS can be served only during non-ABSs, whereas UEs of the shared system can be served during ABSs to allow time orthogonality while serving UEs of both systems, which in turn improves the quality of the received signal at UEs of both systems. 

Hence, multiband enabled, DTX power controlled, and ABS-based eICIC technique employed in-building SBSs can be set for optimal performance of both the SE and the EE by reusing multiple spectra to each SBS both horizontally on 2D space on Earth and vertically within any building such that the normalized SE and EE performances of SBSs are equal to each other. The number of times of reusing a spectrum to each SBS both horizontally and vertically, which results in no further improvement in the EE performance, defines the upper limit of reusing the spectrum in terms of *L* to SBSs for a cost-efficient deployment of SBSs for a given value of ζ as shown in Figure 11. 

### 6.6. Performance Comparison 

#### 6.6.1. SE and EE Performances

Table 3 shows SE and EE performances of OSS and nOSS techniques in comparison with 10 times, as well as 100 times, that of 4G mobile systems. It can be found that for certain values of *L*, i.e., the number of times of horizontal spectrum reuses on 2D space on Earth, irrespective of the number of times of vertical spectrum reuses per building, i.e., ζ and application of the DTX power to SBSs, both OSS and nOSS techniques can achieve 10 times, as well as 100 times SE and EE of 4G mobile systems. Further, nOSS requires less number of times of horizontal spectrum reuses *L* than OSS due to vertical reuse of spectrum in inter-floor level more than once per building in nOSS. Furthermore, the value of L^*^, satisfying both SE and EE requirements, is defined solely by the requirement for SE, for both OSS and nOSS, regardless of the value of ζ and the use of the DTX power to SBSs. It is to be noted that, with an application of the DTX power, the energy consumption per bit can be reduced for both OSS and nOSS, irrespective of the value of ζ. 

According to [68,69], it is expected that 5G mobile systems will be able to achieve 10 times average SE (i.e., 27-37 bps/Hz), as well as 10 times average EE (i.e., 3 µJ/b), of 4G systems [68,69]. Assume that, for the beyond 5G (i.e., the sixth-generation (6G)) mobile system, the requirements for an average SE is 10 times (i.e., 270-370 bps/Hz) [70], as well as for an average EE is 10 times (i.e., 0.3 µJ/b) [71], of 5G systems. Hence, based on the above discussion, since both OSS and nOSS can achieve 10 times average SE, as well as average EE, of 5G systems, the proposed techniques can easily satisfy the average SE and average EE requirements for 5G and beyond 5G mobile systems. In summary, the requirement to satisfy both SE and EE can be performed by changing either ζ or *L*, or both. As compared to other domain, space-domain play a significant role in improving both SE and EE, whereas the DTX power has an impact mainly on EE.

#### 6.6.2. Average User Experience Data Rate

According to [22], the average user experience data rate is expected to be 100 Mbps throughout the macrocell coverage for 5G mobile systems. Using nOSS, the average spectrum that can be allocated per indoor UE is given as follows. Total average capacity per 3D cluster of eight SBss or eight UEs that can be achieved for 40 MHz is 1814.6 Mbps for all four transceivers per SBS, resulting in per UE throughput of 28.35 Mbps. In low-frequency bands, due to the scarcity of available spectrum, including the maximum of five component carriers with a total of 100 MHz spectrum in the 2-GHz band, as well as the maximum of 100 MHz spectrum in the 3.5-GHz band that can be allocated for 5G systems to operate in parallel with satellite systems proposed by Federal Communications Commission (FCC) [72], other than employing advanced techniques such as massive multiple-input multiple-output (MIMO), beamforming, and higher modulation and coding schemes (MCSs), it is difficult to achieve 100 Mbps using Single-Input Single-Output (SISO) antenna systems, particularly in indoor environments. However, since for 5G, due to the availability of a considerable amount of mmWave spectrum, more spectrum can be allocated to an operator in mmWave bands. 

In line with this, for example, Japan has recently allocated the maximum of 100 MHz in the 3.5-GHz band and 400 MHz in the 28-GHz mmWave band to each of the four operators to run 5G systems. Further, a large amount of unlicensed spectrum is also available in the 60-GHz band. Hence, for example, if we increase only the spectrum of the 28-GHz band from 10 MHz to about 60 MHz, the per UE throughput becomes 103.43 Mbps, which exceeds the expected requirement for 5G systems. Likewise, we can satisfy per UE throughput requirement for 5G systems by increasing the spectrum of 60-GHz, keeping 28-GHz unchanged. This is because 2-GHz and 3.5-GHz bands are upper limited by available spectrum bandwidth of 100 MHz and are possessing poor channel link performance. Hence, even with applying the maximum of 100 MHz of 2-GHz, or 3.5-GHz, or both, using (12), the expected per UE average throughput of 100 Mbps cannot be obtained irrespective of applying OSS and nOSS techniques. Note that, similar to the average SE and EE requirements for beyond 5G systems, if we assume that the average user experience data rate for beyond 5G, i.e., 6G, systems is expected to be 10 times of 5G systems (i.e., 1 Gbps) [70], following the above discussion for 5G systems, by increasing the spectra of both 28-GHz and 60-GHz mmWave bands, we can achieve as well per UE throughput requirement of 1 Gbps for the beyond 5G systems. This result also signifies the importance of mmWave bands in 5G and beyond mobile systems due to their good channel link performance within a short distance. 

## 7. Conclusions

In this paper, we have presented a framework that exploits the four most interconnected-domain for spectral and energy efficiencies (including, power, time, frequency, and space) to take advantage of higher degrees of freedom to maximize spectral efficiency and energy efficiency performances using ultra-dense in-building small cells for 5G and beyond mobile systems. In doing so, the proposed framework in each domain has been described comprehensively both qualitatively and analytically. We have then derived system-level average capacity, spectral efficiency, and energy efficiency performance metrics, as well as defined the condition for optimality for both spectrum and energy efficiencies. Besides, an algorithm for the proposed framework has been developed. Extensive system-level performance evaluation has been carried out to show the impact of each domain on spectral and energy efficiencies, as well as to define the condition for optimality of a cost-efficient small cell deployment. Finally, we have compared the performances of the proposed framework in terms of spectral efficiency, energy efficiency, as well as average user experience data rate with the corresponding requirements for 5G and beyond mobile systems. 

It has been found that both EE and SE can be improved by exploiting all four domains of the proposed framework. More specifically, firstly, by exploiting frequency-domain, as compared to a single-band, multiband enabled SBSs can achieve higher SE and EE. Secondly, by exploiting spatial-domain in a multistory building, the same set of spectra can be reused to both horizontally and vertically separated multiband enabled SBSs more than once to improve both SE and EE. Thirdly, by exploiting the power-domain, the SBS can be turned on and off based on the traffic demand using the DTX power mechanism to reduce the power consumption, resulting in an improvement in energy efficiency. Fourthly, by exploiting the time-domain ABS-based eICIC technique, CCI originated due to sharing the spectrum of another system with multiband enabled SBSs, can be avoided to improve the quality of the received signal at UEs of both systems. Further, it has been shown that the optimal performance of both the SE and the EE can be set by reusing spectra to each SBS within any building such that the normalized SE and EE performances of SBSs are equal to each other. Furthermore, it has been found that, for a cost-efficient deployment of SBSs, an upper limit for reusing the same spectrum in a building can be defined in terms of horizontal spatial reuse of spectrum L such that any further spectrum reuse to SBSs both horizontally and vertically results in no further improvement in EE for a given value of vertical spatial reuse of spectrum ζ. Hence, depending on the environmental and building profiles of a certain area, either horizontal or vertical, or both spatial reuse of spectrum can be exploited to achieve both SE and EE targets.

Lastly, 5G and beyond mobile systems are needed to be multiband enabled mobile systems, including both microwave and mmWave spectra, where a microwave spectrum such as 2-GHz can be employed to provide large outdoor macrocell coverage, and mmWave spectra such as 28-GHz and 60-GHz bands along with microwave spectra such as the 2-GHz and FCC recommended 3.5-GHz bands can be employed in indoor environments to address a large volume of high data rate demand of indoor UEs, as well as the envisioned high system-level spectral and energy efficiencies. 

## Figures and Tables

**Table 1:** Parameters and values of the CIF path loss model.

Environment	*n*	*b*	*f*_0_ (GHz)	Δ (dB)
LOS	2.1	0.32	51	9.9
NLOS	3.4	0.22	49	11.9

**Table 2:** Default parameters and assumptions.

Parameters and Assumptions					Value		
E-UTRA simulation case^1^					3GPP case 3		
Cellular layout^2^, Inter-site distance (ISD)^1,2^, transmit direction					Hexagonal grid, dense urban, 3 sectors per macrocell site, 1732 m, and downlink		
Carrier frequency^2,3^		2-GHz for licensed MNO 1, 28-GHz for licensed MNO 2, 3.5-GHz (for licensed satellite spectrum), 60-GHz line-of-sight (for unlicensed spectrum)					
System bandwidth		10-MHz for each licensed spectrum as well as the unlicensed spectrum					
Number of cells		1 macrocell, 2 picocells, 64 SBSs per building for MNO 1					
Total BS transmit power^1^ (dBm)		46 for macrocell^1,4^, 37 for picocells^1^, 20 (for 2-GHz and 3.5 GHz), 19 (for 28-GHz), and 17.3 (for 60-GHz unlicensed spectrum) for FBS^1,3,4,6^					
Co-channel fading model^1,5,6^		Frequency selective Rayleigh for the MBS and picocell BS (PBSs), Rician for FBSs (for 2-GHz and 3.5-GHz), and no small-scale fading effect for LOS 28-GHz and LOS 60-GHz					
External wall penetration loss^1^ (*L*_ow_)						20 dB (for 2-GHz and 3.5 GHz)	
Path loss	MBS and a UE^1^				Outdoor macrocell UE	*PL*(dB) = 15.3 + 37.6log_10_*R*, *R* is in m	
					Indoor macrocell UE	*PL*(dB) = 15.3 + 37.6log_10_*R* + *L*_ow_, *R* is in m	
	PBS and a UE^1^				*PL*(dB) = 140.7+36.7log_10_*R*, *R* is in km		
	SBS and a UE^1,2,3,5^				*PL*(dB) = 127+30log_10_(*R*/1000), *R* in m (for 2-GHz and 3.5 GHz), $PL[\mathrm{dB}]=61.4+10n\left(1+b\left(f-f_{0}/f_{0}\right)\right)\log_{10}\left(d/d_{0}\right)+X_{\Delta }$ (for 28-GHz LOS where *d*_0_ = 1 m, *n* = 2.1, *b* = 0.32, and *f*_0_ = 51-GHz), and *PL*(dB)=68+21.7log_10_(*R*), *R* in m (for 60-GHz)		
Lognormal shadowing standard deviation (dB)			8 for MBS^2^, 10 for PBS^1^, and 10 for 2-GHz and 3.5-GHz spectrum, 9.9 for LOS 28-GHz spectrum, and 0.88 for LOS 60-GHz spectrum for FBS^2,3,5^				
Antenna configuration					Single-input single-output for all BSs and UEs		
Antenna pattern (horizontal)				Directional (120^0^) for MBS^1^, omnidirectional for PBS^1^ and SBS^1^			
Antenna gain plus connector loss (dBi)						14 for MBS^2^, 5 for PBS^1^, 5 for SBS^1,3,6^	
UE antenna gain^2,3,6^					0 dBi (for 2-GHz and 3.5-GHz spectrum), 5 dBi (for 28-GHz and 60-GHz spectrum, Biconical horn)		
UE noise figure^2,6^ and UE speed^1^					9 dB (2-GHz and 3.5 GHz) and 10 dB (for 28-GHz and 60-GHz), 3 km/hr		
Total number of macrocell UEs and small cell UEs per building for MNO 1						30 and 64 respectively	
Picocell coverage and macrocell UEs offloaded to all picocells^1^						40 m (radius), 2/15	
Indoor macrocell UEs^1^					35%		
The 3D multistory building, and SBS models (for regular square-grid structure)					Number of buildings		*L*
					Number of floors per building		8
					Number of apartments per floor		8
					Number of SBSs per apartment		1
					SBS activation ratio		100%
					SBS deployment ratio		1
					Total number of SBSs per building		64
					Area of an apartment		10×10 m^2^
					Location of an SBS in an apartment		Center of the ceiling
Scheduler and traffic model^2^					Proportional Fair (PF) and full buffer		
Type of SBSs					Closed Subscriber Group FBSs		
*T*_ABS_ for the satellite system					1/8		
Channel State Information (CSI)					Ideal		
TTI^1^ and scheduler time constant (*t*_c_)						1 ms and 100 ms	
Total simulation run time					8 ms		

taken ^1^ from [65], ^2^ from [63], ^3^ from [66], ^4^ from [67], ^5^ from [55], ^6^ from [64].

**Table 3:** Comparison of average SE and average EE performances of OSS and nOSS techniques. with that expected for 5G and beyond mobile systems.

Space-Domain	Power-Domain	Performance Metrics	Frequency-and Time-Domain	
			L^*^ (corresponding value of SE or EE)	
Technique	SBS Power Management Strategy		100 Times of 4G Systems (for beyond 5G, i.e., 6G, Systems)	10 Times of 4G Systems (for 5G Systems)
**OSS (** ζ ** = 1)**	With DTX	EE	1 (0.19 µJ/b)	1 (0.19 µJ/b)
		SE	27 (378.3 bps /Hz)	3 (42.4 bps /Hz)
		Both EE and SE	27	3
	Without DTX	EE	1 (0.25 µJ/b)	1 (0.25 µJ/b)
		SE	27 (378.3 bps/Hz)	3 (42.4 bps /Hz)
		Both EE and SE	27	3
**nOSS (** ζ ** = 6)**	With DTX	EE	1 (0.044 µJ/b)	1 (0.044 µJ/b)
		SE	5 (378 bps/Hz)	1 (76 bps/Hz)
		Both EE and SE	5	1
	Without DTX	EE	1 (0.047 µJ/b)	1 (0.047 µJ/b)
		SE	5 (378 bps/Hz)	1 (76 bps/Hz)
		Both EE and SE	5	1
**nOSS (** ζ **= 20)**	With DTX	EE	1 (0.0194 µJ/b)	1 (0.0194 µJ/b)
		SE	2 (504 bps/Hz)	1 (250.2 bps/ Hz)
		Both EE and SE	2	1
	Without DTX	EE	1 (0.022 µJ/b)	1 (0.022 µJ/b)
		SE	2 (504 bps/Hz)	1 (250.2 bps/ Hz)
		Both EE and SE	2	1
